# Immunization against ROS1 by DNA Electroporation Impairs K-Ras-Driven Lung Adenocarcinomas

**DOI:** 10.3390/vaccines8020166

**Published:** 2020-04-06

**Authors:** Federica Riccardo, Giuseppina Barutello, Angela Petito, Lidia Tarone, Laura Conti, Maddalena Arigoni, Chiara Musiu, Stefania Izzo, Marco Volante, Dario Livio Longo, Irene Fiore Merighi, Mauro Papotti, Federica Cavallo, Elena Quaglino

**Affiliations:** 1 Department of Molecular Biotechnology and Health Sciences, University of Torino, 10126 Torino, Italy; federica.riccardo@unito.it (F.R.); giuseppina.barutello@unito.it (G.B.); angelapetito@hotmail.it (A.P.); lidia.tarone@unito.it (L.T.); laura.conti@unito.it (L.C.); maddalena.arigoni@unito.it (M.A.); chiara.musiu@gmail.com (C.M.); irene.merighi@unito.it (I.F.M.); 2 Department of Oncology, University of Torino, 10043 Orbassano, Italy; stefaniaizzo_1990@libero.it (S.I.); marco.volante@unito.it (M.V.); mauro.papotti@unito.it (M.P.); 3 Institute of Biostructures and Bioimaging (IBB), Italian National Research Council (CNR), 10126 Torino, Italy; dariolivio.longo@cnr.it

**Keywords:** DNA plasmid electroporation, ROS1, NSCLC

## Abstract

Non-small cell lung cancer (NSCLC) is still the leading cause of cancer death worldwide. Despite the introduction of tyrosine kinase inhibitors and immunotherapeutic approaches, there is still an urgent need for novel strategies to improve patient survival. ROS1, a tyrosine kinase receptor endowed with oncoantigen features, is activated by chromosomal rearrangement or overexpression in NSCLC and in several tumor histotypes. In this work, we have exploited transgenic mice harboring the activated K-Ras oncogene (K-Ras^G12D^) that spontaneously develop metastatic NSCLC as a preclinical model to test the efficacy of ROS1 immune targeting. Indeed, qPCR and immunohistochemical analyses revealed ROS1 overexpression in the autochthonous primary tumors and extrathoracic metastases developed by K-Ras^G12D^ mice and in a derived transplantable cell line. As proof of concept, we have evaluated the effects of the intramuscular electroporation (electrovaccination) of plasmids coding for mouse- and human-ROS1 on the progression of these NSCLC models. A significant increase in survival was observed in ROS1-electrovaccinated mice challenged with the transplantable cell line. It is worth noting that tumors were completely rejected, and immune memory was achieved, albeit only in a few mice. Most importantly, ROS1 electrovaccination was also found to be effective in slowing the development of autochthonous NSCLC in K-Ras^G12D^ mice.

## 1. Introduction

Lung cancer is still one of the major unresolved issues in the oncology panorama [1]. Overall, around 80%–85% of lung cancer cases can be classified as non-small cell lung cancer (NSCLC) [2,3]. The best option for long-term survival is surgery when NSCLC is diagnosed in its early stages and is resectable [4,5,6]. Unfortunately, the five-year survival is poor even in this case, although there is little improvement if patients are treated with neoadjuvant and/or adjuvant chemotherapy [7,8,9,10]. Instead, when NSCLC is diagnosed in advanced or metastatic stages, which occurs in more than 70% of patients, a dramatically worse prognosis is observed, with a 5-year survival of less than 10% when using conventional therapeutic options, including a combination of chemotherapy and radiotherapy [5,11]. Significant improvements in overall survival have been observed in advanced NSCLC patients treated with checkpoint inhibitors (CIs), such as monoclonal antibodies (mAbs) that target PD-1 (nivolumab and pembrolizumab) and PD-L1 (atezolizumab), compared to patients treated with chemotherapy and radiotherapy alone [12,13,14,15]. However, there is great heterogeneity in the patient response to therapy, including acquired resistance [16], and complete responses are rare [6,17,18]. Moreover, patients often experience significant immune-related adverse effects [19,20].

In addition to CIs, molecular-targeted therapies against the most common NSCLC drivers (the mutated epidermal growth factor receptor, EGFR, and the translocated anaplastic lymphoma kinase, ALK) have been developed and clinically approved [21,22,23,24]. Although effective tumor shrinking and disease control have been observed, acquired resistance is still an unavoidable problem and new approaches that target these molecules are needed.

Another important issue in the management of advanced NSCLC is to expand the spectrum of subtypes that are eligible for targeted therapy by identifying other driver alterations besides the already known EGFR and ALK. With this in mind, we have focused on the c-ros oncogene 1 receptor tyrosine kinase (ROS1), an orphan tyrosine kinase receptor whose aberrant activation has been implicated with enhanced tumor cell growth, proliferation, metastasization, and resistance to chemotherapy [25,26,27]. The most common activating alterations of ROS1 in NSCLC are gene fusion with the solute carrier family 34 member 2 (SLC34A2) and CD74, as well as protein overexpression, gene amplification and mutations [28,29,30,31]. Although the reported frequency of these alterations in an unselected NSCLC population is estimated to be <3% [32,33,34], they rarely overlap with EGFR mutations and ALK fusions [35] and are prevalent in young, never-smoker patients [36,37], defining ROS1-positive NSCLC as a unique subset of patients with a potentially targetable driver oncogene.

The proven existence of an evolutionary link between ROS1 and ALK receptors [38,39] has led to the Food and Drug Administration (FDA) approval of the ALK inhibitor crizotinib [39,40] for the treatment of advanced ROS1-rearranged NSCLC in 2016 [41,42] and finally to its recommendation as standard-of-care for advanced NSCLC patients with known ROS1 alterations [43]. Unfortunately, as it has already been described for ALK-positive NSCLC patients, resistance to crizotinib occurs as a consequence of secondary mutations in the ROS1 tyrosine kinase domain [44]. 

The immune targeting of tyrosine kinases is a promising alternative, or complement, to tyrosine kinase inhibitor-based targeted therapies [45,46]. When tyrosine kinases, such as ROS1, are expressed on the plasma membrane, their immune targeting can either be achieved via the passive administration of mAbs or by actively immunizing the patient against the tyrosine kinase. The potential of the passive administration of mAbs against different tumor associated antigens [47,48] has been demonstrated by several approved clinical protocols. However, mAbs are biologic products, with a short-lived therapeutic action, that can elicit a number of adverse events [49]. Among them, mAb-related infusion problems, sometimes associated with acute hypersensitivity [50], may arise. Other than the well-known dermatologic and cardiac toxicities [51], a number of gastrointestinal, endocrine, and other inflammatory reactions have been described, which are related to the unbalance between immune recognition and tolerance [49]. Moreover, the development by the patient’s immune system of neutralizing antibodies directed against the mAbs and the possible tumor antigen mutation occurring in the epitope recognized by the mAbs are responsible for the tumor relapse observed in most treated patients. In this contest, the concomitant induction of a polyclonal antibody and T-cell response induced by active immunization through therapeutic vaccines offers the possibility to overcome these issues. Moreover, the specific long-lasting immunological memory elicited by the patient’s own immune system could limit the number of treatments needed for each patient and, most importantly, the side effects associated with mAb administration.

The endowed properties of ROS1, i.e., its prevalent expression in cancer cells, in which it plays a clear key oncogenic role, and its limited expression on healthy tissues, make it an ideal oncoantigen to target with vaccination [52]. Among the vaccination strategies used until now, gene-based vaccines have been shown to possess a number of advantages compared to cell- and protein/peptide-based vaccines, being a relatively simple and very flexible way of activating the immune response in animal models against a specific antigen [53,54,55]. However, simple naked plasmid DNA injection is accompanied by limited uptake and a consequent inadequate antigen transcription by transfected cells [56], and this is particularly evident when naked DNA vaccines are scaled up from smaller species such as mice and rats to non-human primates and humans [57,58]. This issue can be overcome through the use of the in vivo intramuscular electroporation of DNA plasmids (electrovaccination) that has been one of the most effective strategies among the innovative delivery methods exploited until now [59,60,61,62]. The principle behind electrovaccination efficacy is strikingly simple: the brief electrical pulses applied in the site of DNA injection create transient ‘pores’ in the muscle cell membranes that allow the DNA to easily enter into the cell cytoplasm [63]. Immediately following cessation of the electrical field, the pores seal without causing cell death. The increased DNA vaccine cellular uptake leads to an amplified protein expression and to an enhanced immune response against the target antigen [64,65,66]. Moreover, electroporation itself works as an adjuvant, since it induces a local inflammation at the injection site and the generation of a pro-immunogenic microenvironment [66,67]. Several devices that ensure safe, tolerable, reproducible, and clinically acceptable in vivo DNA plasmid electroporation have been introduced [68,69] and, in this regard, the Cliniporator (IGEA Srl, Carpi, Italy) has been approved for human and veterinary clinical practice.

To test the efficacy of active immunotherapy through DNA electrovaccination against ROS1-positive NSCLC, we exploited transgenic mice that harbor a latent oncogenic K-Ras allele, at the endogenous locus, that is capable of spontaneous activation in vivo, due to an activating mutation at codon 12 (K-Ras^G12D^ mice). These mice spontaneously develop NSCLC with 100% of penetrance [70]. In this work, we demonstrated that these mice are a reliable translational model for the study of human NSCLC and for investigating the effects of ROS1 targeting, as ROS1 is expressed at all stages of NSCLC progression. Moreover, we have generated a ROS1-positive cancer cell line, starting from a NSCLC that arose in a K-Ras^G12D^ mouse, that can be serially transplanted into syngeneic mice, offering an additional tool for studying the potential of ROS1 targeting.

Since ROS1 is a self-tolerated antigen in mice and it is impossible to know in advance whether the self-homologous sequence of a given antigen is able to break immune tolerance, we evaluated the anti-tumor efficacy of two different plasmids that code for the optimized sequence of the self-murine ROS1 and for the xenogeneic human ROS1 sequence. Both DNA vaccines proved to be able to successfully immunize mice against ROS1 and impair ROS1-positive NSCLC growth and metastasization.

## 2. Materials and Methods 

### 2.1. Mice

Wild-type (wt) SV129 mice were provided by Charles River Labs (Wilmington, MA, USA). Heterozygous K-Ras^G12D^ mice, on SV129 background, were kindly provided by Dr. G. Lozano (University of Texas, Houston, TX, USA). Mice were maintained in the animal house at the Molecular Biotechnology Center (University of Torino, Torino, Italy) under a 12-h light–dark cycle and provided with food and water ad libitum. Heterozygous K-Ras^G12D^ male mice were mated with wt female mice. Female and male pups were genotyped as previously described [70]. Briefly, PCR analysis was performed on tail DNA obtained at weaning using the following primers (from Invitrogen, Carlsbad, CA, USA): a 5′ forward wt (5′-TGCACAGCTTAGTGAGACCC-3′) and a 3′ reverse wt (5′-GACTGCTCTCTTTCACCTCC-3′) specific for the amplification of the K-Ras wt allele (amplification band 220 bp) with the addition of a competitive 3′ reverse mutated primer (5′-GGAGCAAAGCTGCTATTGGC-3′) specific for the G12D substitution, giving rise to a 390 bp amplified band in the presence of the mutation. The resulting PCR products were run on a 1.5% agarose gel to discriminate the presence of the mutated K-Ras^G12D^ allele in heterozygosis.

Individually tagged mice of the same age were treated in conformity with national and international regulations and policies, and all animal experiments were approved by the Faculty Ethical Committee and by the Italian Ministry of Health (approval number 1146/2015-PR).

### 2.2. Magnetic Resonance Imaging (MRI)

Mice were anesthetized at different weeks of age (10, 20, and 30) via the intramuscular injection of a 40 μL mixture of Zoletil 100 (Virbac, Milano, Italia; 80 mg/Kg) and Rompun (Bayer, Milano, Italy; 16 mg/Kg) diluted in phosphate buffered saline (PBS). Representative MR images of tumor progression in transgenic K-Ras^G12D^ mice were acquired on a Bruker Avance 300 (Bruker, Ettlingen, Germany) operating at 7T with ParaVision 5.1 using a 30 mm insert birdcage. T2w axial, coronal, and sagittal MR images, with an in-plane resolution of 117 μm, were acquired with a breath-triggered sequence respiratory gating to reduce lung movement artifacts, and using Rapid Acquisition with Refocused Echoes (RARE) sequence (typical settings TR/TE/NEX/RARE factor = 4.0 s/34.8 ms/3/16). A 256 × 256 acquisition matrix was used with a field of view of 30 × 30 mm^2^. The slice thickness was 1 mm, and the number of slices was 18 to 20, which was sufficient to cover the entire lung so that the tumor volume could be measured. The T2w sequence displays the tumor location, size, and shape in both the left and right lungs, providing clear boundaries with normal lung tissue. To quantify the tumor volume over time in wt and in ROS1-vaccinated mice, images were acquired on a 1T Aspect M2-High Performance MRI System (Aspect Magnet Technologies Ltd., Netanya, Israel) consisting of an NdFeB magnet, equipped with a 35 mm solenoid Tx/Tr coil. The MR images were acquired with a Fast Spin Echo (FSE) sequence (TR 2800 ms; TE 44 ms; number of slices 18–20; slice thickness 1 mm; FOV 100 × 100 mm; matrix 256 × 256).

### 2.3. Spontaneous Lung Tumor Volume Measurements

The data analysis of MR images was performed using an open-source application, ITK-Snap (http://www.itksnap.org), which allows the segmentation of the lung nodules to be performed in three dimensions, and the calculation of the size of tumor lesions to be carried out [71]. Tumor volume per animal was quantified by calculating the area of visible lung hyperintense regions present in each axial or coronal image sequence slice (usually 18–20 per mouse) and then multiplying the sum of the areas by the slice thickness. The post-processing of the segmented data provided the volume (mm^3^) and displayed the shape of the segmented structure. Tumor volumes were also normalized relative to total lung volumes at the indicated times and expressed as the percentage of lung volume occupied by tumors.

### 2.4. Histopathological and Immunohistochemical Analysis

Tumors and tissues collected from wt and K-Ras^G12D^ mice were fixed in 4% formalin and embedded in paraffin. Sections were stained with hematoxylin and eosin (H&E) for histological evaluation. Immunohistochemical (IHC) staining for ROS1 was performed on 3-μm thick serial paraffin sections using an automated immunostainer (Ventana BenchMark AutoStainer, Ventana Medical Systems, Tucson, AZ, USA) and using a primary anti-ROS1 rabbit mAb (D4D6) (Cell Signaling Technology, Danvers, MA, USA) diluted 1:100. ROS1 scoring was performed in a semiquantitative scale (from 0 to 3) based on the percentage of positive neoplastic cells showing cytoplasmic and/or membrane staining, as follows: 0, no staining; 1+, <10% of positive cells; 2+, 10 to 50% positive cells; 3+, >50% positive cells. IHC staining for PD-L1 was performed in an automated immunostainer (Autostainer Link 48, Agilent Technology, Santa Clara, CA, USA) using the CE IVD diagnostic kit “PDL-1 IHC 22C3 pharmDx”. Results are expressed as the exact percentage of neoplastic cells showing PD-L1 membrane positive staining, irrespective of the staining intensity.

### 2.5. RNA Extraction and qPCR

Normal lung tissues and primary lung adenocarcinomas were collected from wt and K-Ras^G12D^ mice at different stages of cancer progression (corresponding to 10, 20, and 30 weeks of age). Mice were sacrificed via cervical dislocation at the indicated times. Specimens for RNA extraction were stored in RNA later (Sigma-Aldrich, St. Louis, MO, USA) at 4 °C for 24 h and then snap-frozen in liquid nitrogen and stored at −80 °C until use. Total RNA was isolated using an IKA-Ultra-Turrax^®^ T8 homogenizer (IKA-Werke, Staufen im Breisgau, Germany) and TRIzol^®^ reagent (Invitrogen), according to the manufacturer’s instructions. Genomic DNA contamination was removed from total RNA using a DNA-free kit (Ambion, Warrington, UK) as per the manufacturer’s instructions. Total RNA concentration and purity were assessed using a NanoDROP 2000 Spectrophotometer (ThermoFisher, Waltham, MA, USA). RNA quality was evaluated on an Agilent 2100 Bioanalyzer, according to the manufacture’s recommendations (Agilent Technologies). Total RNA was stored at −80 °C until use. DNase-treated RNA (1 μg) was retrotranscribed with RETROscript reagents (Ambion), and qPCRs were carried out using gene-specific primers (QuantiTect Primer Assay), SYBR green, and a 7900HT RT-PCR system (Applied Biosystems, Foster city, CA, USA). Quantitative normalization was performed on the expression of the housekeeping glyceraldehyde 3-phosphate dehydrogenase (GAPDH) and 18s ribosomal RNA genes. Relative gene expression levels were calculated using the comparative ΔCt method [72].

### 2.6. Cell Line

KL-ROS1 is a cell line that we established in vitro from a lung carcinoma that arose spontaneously in a 40-week-old K-Ras^G12D^ female mouse. Cells are cultured in Dulbecco Modified Eagle’s Medium (DMEM) supplemented with 20% heat-inactivated fetal bovine serum (FBS, Sigma-Aldrich) and routinely checked for Mycoplasma contamination using the Mycoalert Detection Kit (Lonza, Zurich, Switzerland) and consistently found to be negative. In order to test ROS1 expression, KL-ROS1 cells were washed in PBS supplemented with 0.2% bovine serum albumin (BSA) and 0.01% sodium azide (Sigma-Aldrich) and stained for ROS1 after fixation and permeabilization with the Cytofix/Cytoperm Fixation/Permeabilization Kit (BD Biosciences, Allschwil, Switzerland). In order to detect the ROS1 positivity, a primary rabbit anti-ROS1 (D4D6) mAb (Cell Signaling Technology) and a secondary fluorescein isothiocyanate (FITC)-conjugated goat anti-rabbit immunoglobulins (Dako, Santa Clara, CA, USA) were used. For transplantable tumor experiments, wt mice were challenged subcutaneously with different doses of KL-ROS1 cells, ranging from 10^5^ to 10^6^. The tumor dimensions of the challenged mice were inspected weekly by palpation. Progressively growing masses >1 mm in diameter were regarded as tumors. Mice were sacrificed when the tumor exceeded 10 mm in diameter.

### 2.7. Flow Cytometry Analysis

In order to characterize the phenotype of splenocytes (SPC) and tumor-infiltrating immune cells, single-cell suspensions were derived from the spleens and from lungs of wt tumor-bearing and K-ras^G12D^-vaccinated mice, as described in [73]. Cells were treated with Fc receptor blocker (BD Biosciences) and then stained for 30 min at 4 °C with the following antibodies: anti-CD45-VioGreen, anti-CD3-FITC, anti-CD4-APC/Vio770, anti-CD8-VioBlue, anti-CD49b-PE, anti-TCRγδ-PE/Vio770, anti-PD1-APC, anti-CD11b-FITC, anti-F4/80-PE/Vio770, anti-Ly6C-APC/Vio770, anti-Ly6G-VioBlue, anti-MHCII-APC (Miltenyi Biotec Bergisch Gladbach, Germany), anti-CD206-PE, and anti-CD69-PE/Vio770 (Biolegend, San Diego, CA, USA). Labeled samples were acquired on a BD FACSVerse and analyzed using FlowJO10.5.3 software.

### 2.8. Mice Immunization through DNA Plasmid Electroporation

The plasmids coding the extracellular and transmembrane domain of the mouse (m) and the human (h) ROS1 cDNA sequences were obtained from GenScript (Piscataway, NJ, USA). Regarding the mROS1 plasmid, the cDNA sequence inserted was optimized in order to enhance its immunogenicity in mice. The ROS1 protein sequence coded by the hROS1 plasmid shows a homology of 80% with the mROS1 protein. Both sequences were cloned in pVAX1 plasmid (Invitrogen), sequenced (BMR Genomics, Padova, Italy), and amplified with EndoFree Plasmid Giga Kits (Qiagen Inc., Hilden, Germany). Before each immunization, mice were anaesthetized, as described above, and then their quadriceps muscle was injected with 50 μg of either pVAX, or mROS1 or hROS1 plasmids diluted in 20 μL of saline solution. Immediately after the injection, two 25-ms transcutaneous low-voltage electric pulses (amplitude 150 V; interval 300 ms) were administered at the injection site via a multiple-needle electrode connected to a Cliniporator^TM^ (IGEA) [74,75,76]. For transplantable tumor growth experiments, wt mice were immunized twice at 14-day intervals, while, as far as the transgenic mouse model is concerned, 6-week-old K-Ras^G12D^ mice were randomly assigned to pVAX, mROS1, and hROS1 experimental groups and immunized three times at 14-day intervals.

### 2.9. Assessment of Anti-ROS1 Cellular Response by ELISPOT

ELISPOT analysis was performed to detect interferon (IFN)-γ release from the lymphocytes that were collected from vaccinated mice. Specifically, 5 × 10^5^ SPC, collected two weeks after the second electrovaccination of wt mice, were plated in duplicate into nitrocellulose 96-well HTS IP plates (Millipore, Bedford, MA, USA) that had been pre-coated with 5 μg/mL of purified anti-mouse IFNγ antibody (clone R4–6A2, BD Biosciences). SPC were stimulated for 48 h at 37 °C with 15 μg/mL of the predicted H2-D^b^ immunodominant peptide (Pep2, FACENNDFL) (Twin Helix, Rho, MI) derived from both the m and hROS1 protein sequences. The selection of the peptide sequence was based on the online peptide prediction tool SYFPEITHI provided by the University of Tubingen (Tubingen, Germany). Briefly, both the murine and the human ROS1 sequences were analyzed according to the SYFPEITHI epitope prediction algorithm described in [77], in order to identify the predicted H2-D^b^-restricted nonamers. All possible nonamers were listed on the basis of their probability of being processed and presented to T cells, and among them, we selected the highest-scoring peptide shared by both sequences.

SPC stimulated with 2 μg/mL of Concanavalin A (ConA) were used as internal technical controls. Plates were developed according to the manufacturer’s instructions (BDTMELISPOT Set, BD Biosciences).

### 2.10. Statistical Analysis

Statistical differences were evaluated using GraphPad software 6.0 (GraphPad Inc., San Diego, CA, USA). Differences in tumor incidence and mouse survival were analyzed using the Wilcoxon test, while differences in the tumor volumes, in the percentage of volume occupied by the tumor, in qPCR analysis, in anti-ROS1 antibody titers, in the number of IFN-γ-releasing SPC, and in the percentages of the different immune cells were analyzed using either the Student’s t-test or one-way ANOVA.

## 3. Results

### 3.1. K-Ras^G12D^ Transgenic Mice are a Valuable Pre-Clinical Model for Human NSCLC

**Figure 1:**
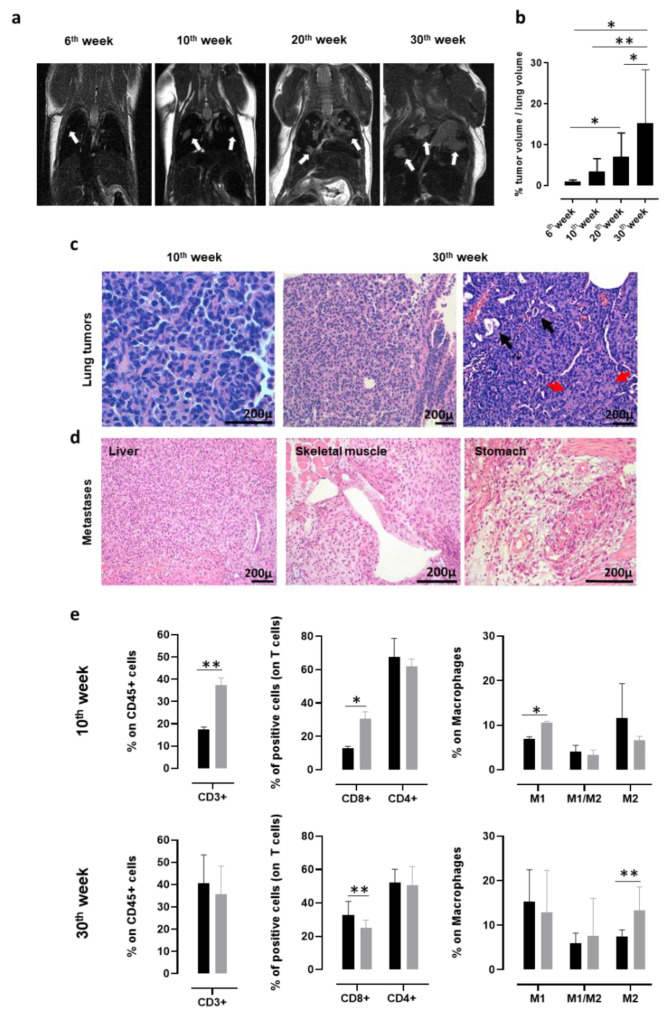
Characterization of lung cancer progression in K-Ras^G12D^ mice. (**a**) Representative MRI images acquired with a 7T high field scanner of K-Ras^G12D^ mice at 6, 10, 20, and 30 weeks of age (N = 3 mice for each time point). White hyperintense regions, indicated by arrows, are tumors. (**b**) Percentage (mean + standard deviation (SD)) of tumor volume occupying the lungs of K-Ras^G12D^ mice (N = 5–16) at indicated weeks of age. Statistically significant differences were calculated using the Student’s *t*-test: * *p* = 0.05; ** *p* = 0.008. (**c**) Representative hematoxylin and eosin (H&E) evaluation of lung sections from K-Ras^G12D^ mice at the initial (10 weeks of age, left panel) and late (30 weeks of age, middle, and right panels) stages of tumor progression. Black arrows indicate some figures of microglandular formation; red arrows indicate collagen deposition in desmoplastic stromal reaction. N = 4 mice were analyzed at indicated time points. (**d**) Representative H&E images of extrathoracic metastatic lesions in the liver, skeletal muscle, and stomach, collected from a 48-week-old K-Ras^G12D^ mouse. N = 4 mice were analyzed. (**e**) Cytofluorimetric analysis of immune infiltrates of the lungs (N = 3–12) from 10 (upper panels) and 30-week-old (lower panels) wt (black bars) and K-Ras^G12D^ (grey bars) mice. Graphs show the percentage ± SD of CD45^+^ cells expressing CD3 (left panels), CD8, and CD4 (middle panels) and of the M1 state (CD11b^+^ f4/80^+^ MHCII^+^ CD206^-^), M2 state (CD11b^+^ f4/80^+^ MHCII^-^ CD206^+^), and M1/M2 transitional state (CD11b^+^ f4/80^+^ MHCII^+^ CD206^+^) macrophages (right panels). Statistically significant differences were calculated using the Student’s *t*-test: * *p* = 0.01; ** *p* < 0.005.

In order to monitor the presence and progression of spontaneous NSCLC that developed in K-Ras^G12D^ transgenic mice with a non-invasive technique, MRI for small rodents was exploited. K-Ras^G12D^ mice were imaged at 6, 10, 20, and 30 weeks of age (Figure 1a). Lung microlesions, which were observed as white opaque hyperintense regions, were already visible in 6-week-old K-Ras^G12D^ mice and increased in number and size in 10, 20, and 30-week-old K-Ras^G12D^ mice. Indeed, the segmentation analysis of the lung nodules in three dimensions clearly indicated a significant increase in the percentage of tumor volume occupying the lungs of 30-week-old K-Ras^G12D^ mice, compared to that of 6, 10, and 20-week-old K-Ras^G12D^ mice (Figure 1b). Histological analyses of the lung sections collected from K-Ras^G12D^ mice confirmed that the white opacities revealed by the MRI analyses corresponded to the small foci of lung carcinoma (Figure 1c). Neoplastic foci consisted of growths of polygonal atypical epithelioid cells arranged in a solid architecture, with occasional gland formation, and a desmoplastic reaction that proves stromal infiltration. Mucin production was very scant or absent. No lepidic growth nor non-invasive neoplastic proliferations were seen. The histological appearance of tumor foci was very similar in the different animals, irrespective of the age of the mice (Figure 1c).

It is worth noting that between the 45th and the 50th week of age, 25% of K-Ras^G12D^ mice develop extrathoracic metastases (Figure 1d) in the liver, skeletal muscle, and stomach, which are the same sites of cancer-cell dissemination found in patients affected by NSCLC [78,79].

Flow cytometry analysis of the lungs from 10- and 30-week-old K-Ras^G12D^ mice were performed to investigate the evolution of the tumor microenvironment during cancer progression, and the results were compared to those obtained from the lungs of age-matched wt mice. At 10 weeks of age, a significant increase in CD3^+^, CD8^+^ T cells, and in type-1 polarized macrophages was observed in the lungs of K-Ras^G12D^ mice, as compared to wt mice (Figure 1e, upper panels), suggesting that a spontaneous antitumor immune response was present in the early stages of NSCLC development. On the other hand, the T cell-mediated anti-tumoral response was decreased later in carcinogenesis progression (30 weeks of age) and was accompanied by a significant increase in type-2 polarized macrophages (Figure 1e, lower panels). This immune phenotype of the K-Ras^G12D^ lungs mirrors the setting of human advanced NSCLC, for which the spontaneous conversion from an anti-tumorigenic to a pro-tumorigenic microenvironment has been described to occur over time [80,81,82]. Moreover, PD-L1 was detectable by means of IHC in all samples tested (data not shown), with a percentage of positive tumor cells ranging from 15% to 40%, similar to what was observed in most human NSCLC [83].

These data further support the high translational value of the K-Ras^G12D^ mouse model.

### 3.2. Autochthonous NSCLC and Extrathoracic Metastases of K-Ras^G12D^ Mice Express the ROS1 Oncoantigen

**Figure 2:**
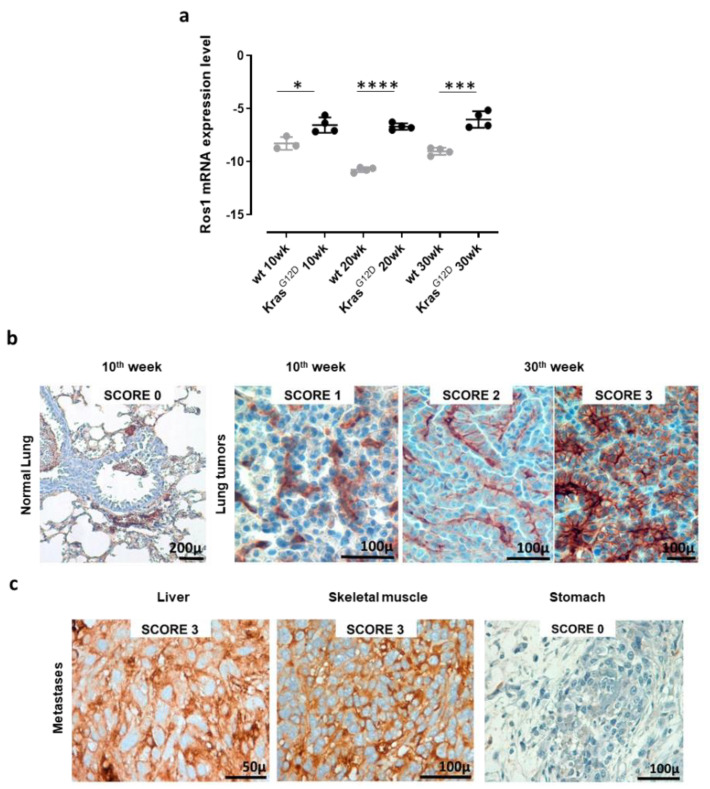
Receptor tyrosine kinase ROS1 expression in lung tumors and metastases from K-Ras^G12D^ transgenic mice. (**a**) qPCR for ROS1 mRNA expression levels in lungs from 10, 20, and 30-week-old wt and K-Ras^G12D^ mice (N = 3–4). The results are expressed as Delta C_T_ (-Dct) between the C_T_ value of the ROS1 gene and the C_T_ value of the GAPDH housekeeping gene. Each dot represents the evaluation of the relative mRNA expression level in a single mouse. Statistically significant differences were calculated using the Student’s *t*-test: * *p* = 0.02; *** *p* < 0.0006; **** *p* < 0.0001. (**b**) Representative images of ROS1 IHC staining of lung samples from a wt (normal lung) mouse, a 10-week-old K-Ras^G12D^ mouse, and two different lung lesions from the same 30-week-old K-Ras^G12D^ mouse. The ROS1 IHC score was attributed as described in the Materials and Methods section. N = 4 mice were analyzed. (**c**) Representative images of the ROS1 IHC staining of metastatic lesions found in a 48-week old K-Ras^G12D^ mouse (liver, skeletal muscle, and stomach). N = 4 mice were analyzed.

To validate the use of K-Ras^G12D^ mice as a model with which to study the effects of ROS1 targeting in NSCLC, we evaluated ROS1 expression in the lungs and extrathoracic lesions of these mice. qPCR analyses of the lungs collected from 10, 20, and 30-week-old K-Ras^G12D^ mice and the corresponding aged-matched wt controls revealed the significant overexpression of ROS1 mRNA in transgenic mice, as compared to controls, at each time point analyzed (Figure 2a and Figure S1). The expression of ROS1 during lung tumorigenesis was also confirmed at the protein level by means of IHC staining (Figure 2b). Indeed, while no staining was observed in the normal lung parenchyma of wt or K-Ras^G12D^ mice, ROS1 protein expression was found in 100% of neoplastic lung lesions from K-Ras^G12D^ mice, although at variable extents and intensities (Figure 2b). It is worth noting that different protein-expression patterns were observed in separate nodules collected from the same lung sample (Figure 2b, middle and right panels), suggesting great intratumor heterogeneity in the lesions.

However, no correlation between ROS1 expression and tumor progression was observed, being both mRNA and protein levels constantly high throughout all the analyzed stages. These data fit well with our previous transcriptional microarray analysis of the lungs of double transgenic K-Ras^G12D^/p53^R172HΔg^ mice [84] from which ROS1 mRNA resulted significantly increased in K-Ras^G12D^/p53^R172HΔg^ double transgenic mice, as compared to wt mice, at the different time points analyzed (Figure S2), despite no correlation between ROS1 expression levels and tumor progression being observed.

Interestingly, when analyzed after IHC staining, metastatic lesions of K-Ras^G12D^ mice showed a heterogeneous pattern of ROS1 protein expression, from 0 in the stomach to 3 in the liver and skeletal muscle (Figure 2c), highlighting the potential role that ROS1 plays as a target for the therapy of both primary tumors and derived metastases.

### 3.3. Anti-ROS1 Electrovaccination Impairs the Growth and Metastasization of ROS1-Positive Transplantable K-Ras^G12D^ NSCLC

**Figure 3:**
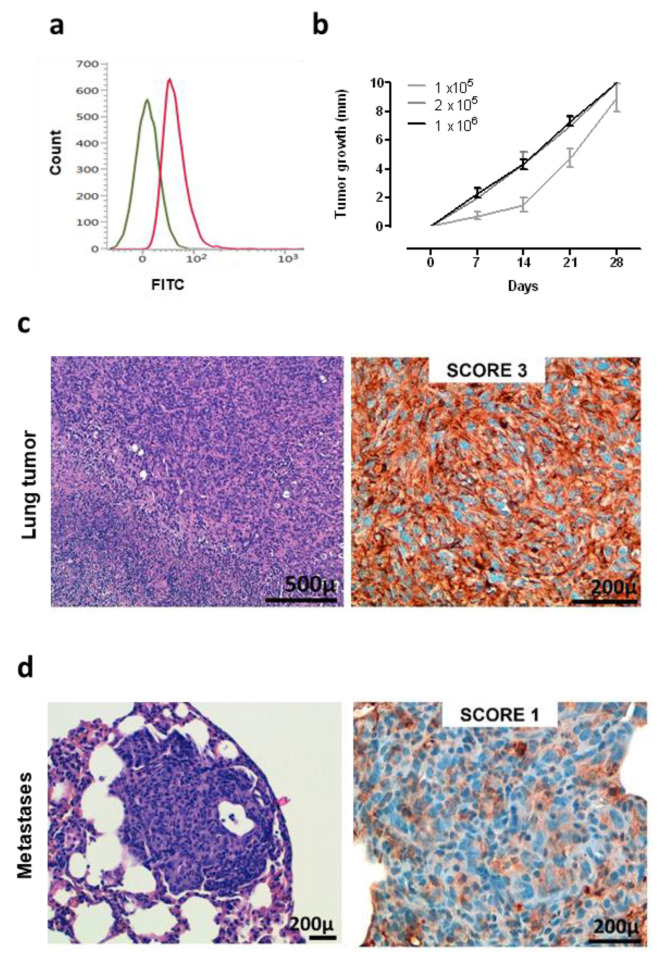
Characterization of KL-ROS1 cell line. (**a**) Representative cytofluorimetric analysis of anti-ROS1 (D4D6) (red line) or control unrelated IgG isotype Ab (green line) staining on KL-ROS1 cells. (**b**) Graph showing the tumor growth of KL-ROS1 cells injected subcutaneously in wt mice at different doses. Data are expressed as mean tumor diameters ± SD for each experimental group (N = 3–5 mice). (**c**,**d**) Representative H&E (left panels) and IHC staining for the ROS1 protein (right panels) of (**c**) an explanted 10 mm mean diameter KL-ROS1-derived tumor and (**d**) a spontaneous KL-ROS1-derived lung metastases. N = 4 mice were analyzed.

A murine NSCLC cell line was generated and established starting from a K-Ras^G12D^ lung tumor. Flow cytometry analysis demonstrated that these cells are ROS1-positive (Figure 3a), and therefore, we called this newly generated cell line KL-ROS1. When injected subcutaneously, KL-ROS1 cells gave rise, in all wt mice, to palpable tumors that reached 10 mm mean diameter in about 30 days (Figure 3b). In accordance with the observations of spontaneous lung tumor samples, IHC staining confirmed that KL-ROS1 tumors do express high levels of the ROS1 protein (Figure 3c), although with heterogeneous intensity (from score 1+ to 3+) in different mice. Similar to the K-Ras^G12D^ mouse model, PD-L1 was co-expressed with ROS1 in KL-ROS1 tumors (data not shown). Moreover, subcutaneous KL-ROS1 tumors spontaneously metastasize to the lungs, and the lung lesions preserve ROS1 overexpression (Figure 3d).

**Figure 4:**
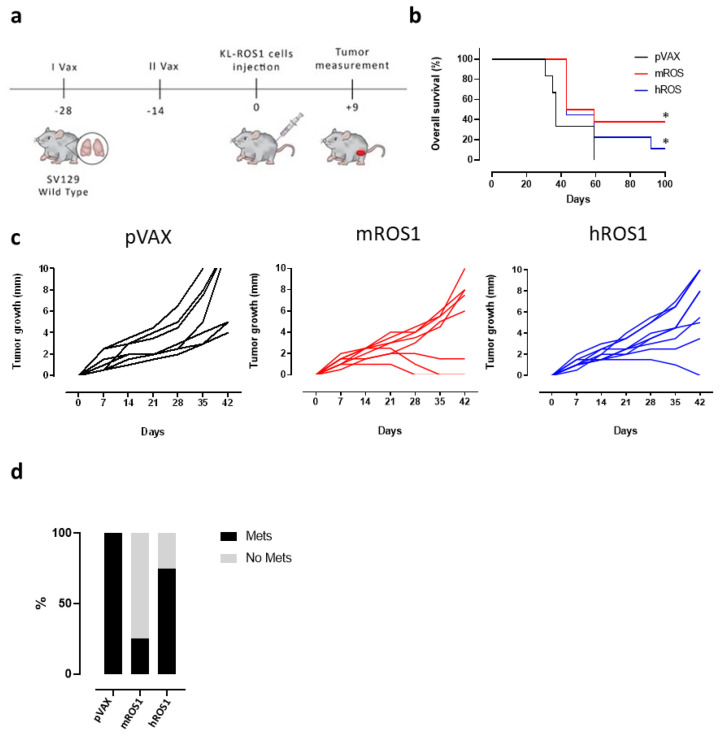
Effect of anti-ROS1 DNA vaccination on transplantable KL-ROS1 tumors. (**a**) Protocol timeline for DNA vaccination through electroporation. (**b**) Overall survival curves of wt mice electrovaccinated with pVAX (black line; N = 7 mice), mROS1 (red line; N = 8 mice), and hROS1 (blue line; N = 9 mice) plasmids. Survival was calculated considering the time (in days) in which KL-ROS1 tumors reached 10 mm in mean diameter. Statistically significant differences in the survival times of m and hROS1-electrovacinated mice, as compared to control mice that were electrovaccinated with pVAX, were analyzed using the Wilcoxon test: * *p* = 0.03, * *p* = 0.05. (**c**) Tumor growth of KL-ROS1 cells injected into pVAX- (black line, left panel, N = 7 mice), mROS1- (red line, middle panel, N = 8 mice), and hROS1- (blue line, right panel, N = 9 mice) electrovaccinated wt mice. Each line represents the growth of a single tumor. (**d**) Percentage of non-metastatic (gray bar) or metastatic (black bar) lungs collected from control, mROS1-, and hROS1-electrovaccinated tumor-bearing mice (N = 4 mice)

To test the potential of ROS1 immune-targeting to inhibit KL-ROS1 tumor growth, we electrovaccinated wt mice twice, at a 14-day interval, with plasmids coding for the extracellular and transmembrane sequence of either the m or hROS1 protein. Two weeks after the second immunization, the animals were challenged subcutaneously with 2 × 10^5^ KL-ROS1 cells (Figure 4a). A significant increase in overall survival was observed in mice vaccinated with mROS1 and hROS1 plasmids, as compared to pVAX controls (Figure 4b). Interestingly, two out of eight mice (25%) vaccinated with mROS1 (Figure 4c, middle panel), and one out of nine mice (11%) vaccinated with hROS1 (Figure 4c, right panel) rejected the tumor. When these tumor-free mice were re-challenged in the opposite flank with the same dose of KL-ROS1 cells, they were protected (not shown), suggesting that anti-ROS1 DNA electrovaccination efficiently induces an immune memory against ROS1 protein-expressing cells.

**Figure 5:**
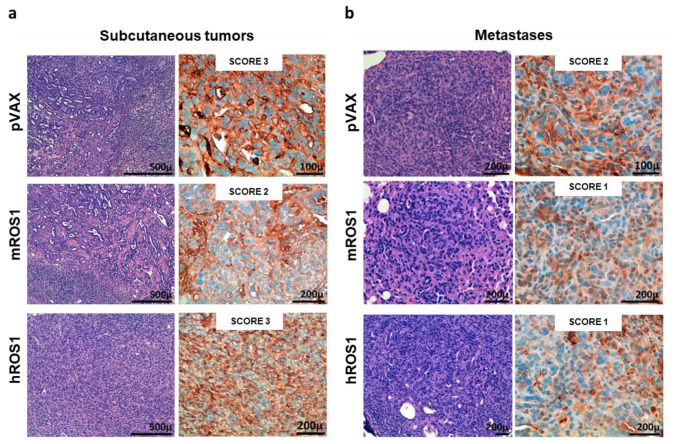
ROS1 expression in transplantable tumors and metastases from wt, and mROS1- and hROS1-vaccinated mice. Representative images of the H&E staining (left panels) and IHC analysis of ROS1 protein expression (right panels) on explanted subcutaneous tumors (**a**) and lung metastases (**b**) from pVAX- (upper panels), mROS- (middle panels), and hROS- (lower panels) electrovaccinated mice. The ROS1 IHC score is indicated in each panel. N = 4 mice were analyzed.

mROS1 and hROS1 vaccination also impacted upon the ability of KL-ROS1 cells to give rise to metastases. Indeed, while 100% of analyzed lungs collected from mice immunized with the pVAX empty vector displayed metastatic lesions, only 25% of mROS1- and 75% of hROS1-electrovaccinated mice showed lung metastases (Figure 4d). Nevertheless, the expression pattern of ROS1 protein was similar in mROS1- and hROS1-vaccinated mice, as compared to control mice vaccinated with the empty pVAX vector, both in subcutaneous and lung lesions (Figure 5). Furthermore, the pattern and the intensity of the PD-L1 expression detectable by IHC did not change in ROS1 electrovaccinated and control mice in all tested samples (data not shown).

Collectively, these data suggest that ROS1 DNA electrovaccination may possess efficacy in hampering tumor growth and metastatic spread.

### 3.4. Immune Response Induced by Anti-ROS1 Electrovaccination

In order to study the mechanisms that may potentially be associated with the impairment of KL-ROS1 tumor growth that was observed in wt mice vaccinated against ROS1, we investigated the immune response induced by the electrovaccination.

Firstly, the presence of anti-mROS1 and anti-hROS1 antibodies was checked in the sera from immunized mice, two weeks after the second electrovaccination. As recombinant m and hROS1 proteins are not commercially available, protein fragments (purity <85%) from the mROS1 (aa 29 to 1038) and hROS1 (aa 28 to 1042 and 1043 to 1859) extracellular domains were used as targets in ELISA. No significant differences were found in the sera from ROS1 and pVAX electrovaccinated mice (Figure S3a). However, when sera from each experimental group were pooled and tested against NIH-3T3 murine fibroblasts transfected with ROS1-coding plasmids, faint binding was evident in the immunofluorescence. In particular, while sera from pVAX electrovaccinated mice did not stain NIH-3T3 cells that were transfected with either mROS1 or with hROS1, those from mice that were electrovaccinated with mROS1 and hROS1 were able to stain the NIH-3T3 cells transfected with mROS1 and hROS1, respectively (Figure S3b). These data indicate that a specific anti-ROS1 antibody response may be induced following ROS1 electrovaccination.

**Figure 6:**
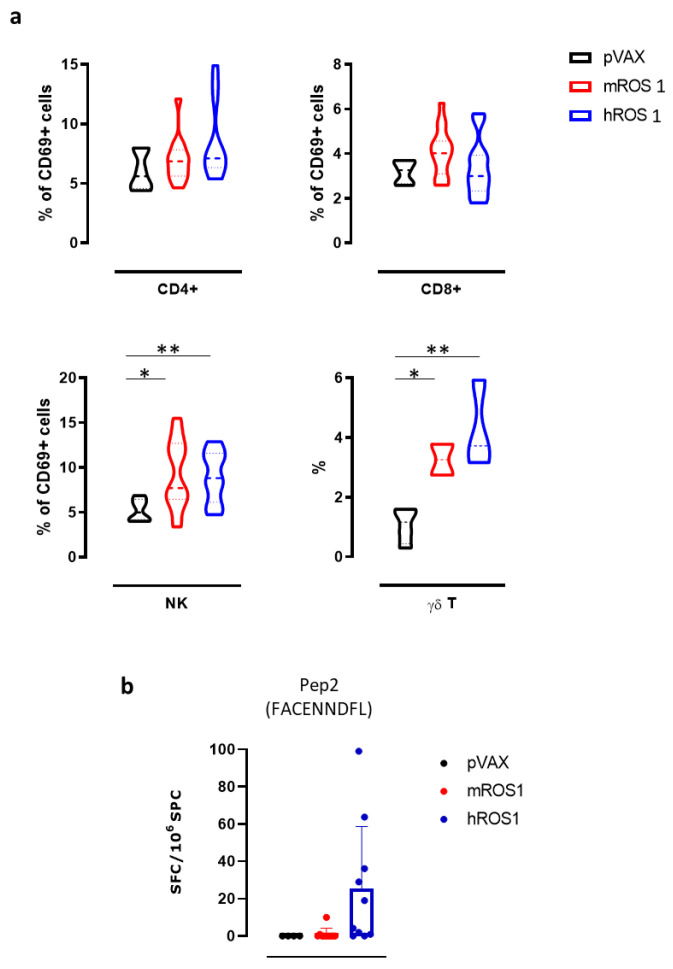
Anti-ROS1 cellular immune response induced by electrovaccination in wt mice. (**a**) Cytofluorimetric analysis of SPC collected from pVAX- (black), mROS1- (red), and hROS1- (blue) electrovaccinated wt mice (N = 6–12). CD45^+^ leukocytes were gated, and CD3^+^ CD4^+^ CD69^+^ cells were identified as activated CD4^+^ T cells, CD3^+^ CD8^+^ CD69^+^ were identified as activated CD8^+^ T cells, CD3^-^ CD49b^+^ CD69^+^ were identified as activated NK cells, and CD3^+^ γδ^+^ were identified as γδ T cells. Results are expressed as a percentage of positive cells using violin plots, which represent the mean and the 95% CI of each group of data. Statistically significant differences in the percentages of γδ T cells and activated CD4^+^ and CD8^+^ T cells, and NK cells in ROS1-electrovacinated and control mice were analyzed using the Student’s *t*-test: * *p* = 0.02; ** *p* < 0.006. (**b**) The detection of the number of IFN-γ-producing cells when SPC that were collected from pVAX- (black; N = 4 mice), mROS1- (red; N = 10 mice), and hROS1- (blue; N = 10 mice) electrovaccinated wt mice were stimulated with the H2-D^b^ immunodominant ROS1-derived peptide (Pep2, FACENNDFL). Results are expressed as spot-forming cells (SFC) per million of SPC plated ± SD and were analyzed using the Student’s *t*-test.

Then, we evaluated the effects of ROS1 electrovaccination on the frequency of various immune cell populations in the SPC of treated mice, using flow cytometry. While no differences were found in the percentage of CD4^+^ T, CD8^+^ T, and natural killer (NK) cells (data not shown), the percentage of NK cells that expressed the activation marker CD69 in SPC from both m and hROS1 electrovaccinated mice was significantly increased compared to controls (9.3 ± 3.8; 9.0 ± 2.8; 5.2 ± 1.2; Student’s *t*-test: * *p* = 0.02; ** *p* < 0.006). Moreover, the frequency of γδ T cells in SPC from both m and hROS1 was twice that found in pVAX electrovaccinated mice (Figure 6a), (3.3 ± 0.7; 4.3 ± 1.5; 1.1 ± 0.6; Student’s *t*-test: * *p* = 0.02; ** *p* < 0.006).

In order to evaluate the ability of ROS1 electrovaccination to induce a ROS1-specific CD8^+^ T cell response, SPC from electrovaccinated mice were tested in an IFN-γ ELISPOT assay after in vitro restimulation with a synthetic nonamer peptide (FACENNDFL) that is shared by m and hROS1 proteins and predicted to be properly presented on H-2D^b^ molecules. Specific IFN-γ-releasing T cells were only found in the SPC from one mROS1 and a few hROS1 electrovaccinated mice (Figure 6b), although no significant differences were observed among groups.

Taken together, these results suggest that ROS1 electrovaccination is potentially immunogenic in mice as demonstrated by the increase of activated NK cells and in the frequency of γδ T cells and by the induction of IFN-γ releasing CD8^+^ T cells in some vaccinated mice. These cells might contribute, together with antibodies and other immune mechanisms that we have not yet explored, to the significant increase in overall survival and to tumor rejection that was observed in immunized mice.

### 3.5. Anti-ROS1 DNA Vaccination Impairs the Development of Spontaneous Lung Cancers in K-Ras^G12D^ Transgenic Mice

**Figure 7:**
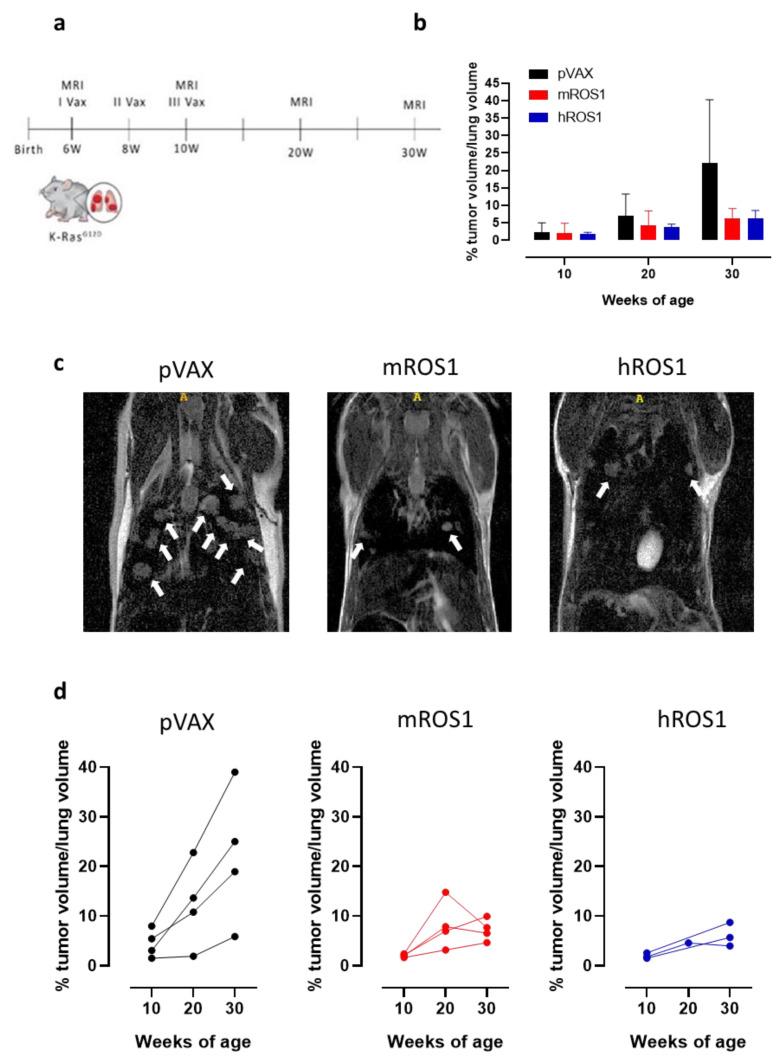
Impact of anti-ROS1 DNA vaccination on the autochthonous lung carcinogenesis of K-Ras^G12D^ mice. (**a**) Protocol timeline for DNA electrovaccination. (**b**) Percentage (mean ± SD) of tumor volume occupying the lungs in 10, 20, and 30-week-old pVAX (black bars), mROS1 (red bars), and hROS1 (blue bars) electrovaccinated K-Ras^G12D^ mice (N = 4–12). (**c**) Representative MRI images of 30-week-old K-Ras^G12D^ mice immunized with pVAX (left panel), mROS1 (middle panel), and hROS1 (right panel) acquired with a 7T high field scanner (N = 3 mice for each time point). White hyperintense regions indicated by arrows are tumors. (**d**) Tumor progression of pVAX- (black bars; N = 4 mice), mROS1- (red bars; N = 4 mice), and hROS1- (blue bars; N = 3 mice) electrovaccinated K-Ras^G12D^ mice over time. Each single mouse was imaged by MRI at 10, 20, and 30 weeks of age. Data are shown as percentage of tumor volume occupying the lungs of each single mouse.

In order to evaluate whether anti-ROS1 DNA electrovaccination can also impair aggressive K-Ras^G12D^-driven autochthonous ROS1-positive lung carcinogenesis, 6-week-old K-Ras^G12D^ mice were electrovaccinated three times, at two-week intervals, with either the mROS1 plasmid, hROS1 plasmid, or the control empty vector pVAX (Figure 7a). Since the percentage of the lung volume occupied by tumors is very similar among K-Ras^G12D^ mice imaged at 6 weeks of age (from 0.375% to 1.389%; Figure 1a,b), animals were randomly distributed in the different immunization groups. The presence of lung adenocarcinomas was monitored and quantified at 10, 20, and 30 weeks of age by MRI.

All vaccinated mice developed lung tumors as expected in this very aggressive model. However, at 30 weeks of age, a clear reduction in the lung volume occupied by the tumors was observed in ROS1-electrovaccinated mice, compared to the age-matched controls (Figure 7b). Lung lesions were fewer and smaller in most of the ROS1 electrovaccinated mice than in the age-matched controls (Figure 7c). In those K-Ras^G12D^ electrovaccinated mice in which it was possible to monitor the lung tumor progression by MRI over time, lower growth speed was observed in all m and hROS1 electrovaccinated compared to control mice (Figure 7d). Notably, a decrease in the total tumor volume was observed between the 20th and the 30th week of age in 2 out of 4 mROS1 and in 1 out of 3 hROS1 electrovaccinated mice (Figure 7d).

Overall, these results suggest that ROS1 electrovaccination may play a role in controlling the growth of the very aggressive autochthonous NSCLC of K-ras^G12D^ mice.

In order to monitor the vaccine-induced immune response, sera from K-Ras^G12D^ mice were tested in ELISA using mROS1 (aa 29 to 1038) and hROS1 (aa 28 to 1042 and 1043 to 1859) protein fragments. Similarly to observations in wt mice, no differences in anti-ROS1 antibody levels were detected (Figure S4a). When the tumor infiltrating cells from 30-week-old K-ras^G12D^ mice were studied by flow cytometry, a slight increase in the percentage of CD4^+^ T and NK cells that expressed the CD69 activation marker, and a slight increase in the γδ T cells was found in m and hROS1 electrovaccinated mice, compared to controls (Figure S4b). As far as PD-L1 expression is concerned, no differences in the percentage of PD-L1-positive cells were observed in the lung lesions of ROS1 electrovaccinated and control K-ras^G12D^ mice (data not shown).

## 4. Discussion

Lung cancer is the leading cause of cancer mortality [85], with NSCLC accounting for approximately 85% of cases. As the earlier stages are relatively asymptomatic, many patients present advanced disease and metastases at diagnosis. The unfeasibility of tumor resection in the majority of cases and the metastatic spread of the disease lead to severe complications, which usually precede the fatal stages by a few months, and conventional therapies (i.e., chemotherapy and radiotherapy) are relatively ineffective. For this reason, NSCLC still remains a hot, unmet topic in the oncology panorama, prompting research toward the development of novel and more effective therapies.

Several genomic NSCLC subsets have been identified, progressively modernizing treatment strategies toward “personalized medicine”. Moreover, novel molecular aberrations, beside those of EGFR and ALK proteins, have been investigated and characterized, with chromosomal rearrangements and the overexpression of ROS1 gaining a great deal of interest [86,87,88]. Constitutively active ROS1 tyrosine kinase has been proven to be a potent tumorigenic driver that activates downstream oncogenic signals, leading to increased cell proliferation and survival. In fact, crizotinib has been FDA-approved for the treatment of ROS1-positive NSCLC, increasing patient survival [40]. However, as for other tyrosine kinase inhibitors, the majority of patients progressively develop resistance to crizotinib [89,90,91]. Herein, we propose using active immunotherapy to induce the patient’s own immune system to develop a long-lasting anti-tumor immune response against ROS1, as an alternative treatment. To this end, in this work, we have exploited two murine models of ROS1-positive NSCLC, the KL-ROS1 cell line and the transgenic K-Ras^G12D^ mice, to test the efficacy of DNA electrovaccination against ROS1. The KL-ROS1 cell line was derived from a lung lesion in a K-Ras^G12D^ mouse. When injected subcutaneously into syngeneic mice, KL-ROS1 cells gave rise to fast-growing ROS1-positive tumors in the site of injection and to lung metastases. Transgenic K-Ras^G12D^ mice spontaneously develop NSCLC early in their life, and about 25% of them display extrathoracic metastases when they succumb for the disease at 11–12 months of age. We detected ROS1 overexpression at both mRNA and protein levels in the primary lung lesions during all the stages of cancer progression and in the extrathoracic metastases developed in several organs of K-Ras^G12D^ mice. By contrast, ROS1 was not detected in normal lung tissue, mirroring the human situation in which ROS1 is not, or is just barely, expressed in healthy lung tissue, bronchial basal cells, peri-bronchial glands, and smooth muscle cells [92]. Similarly, cancer cells were ROS1-positive in the metastatic sites.

Plasmids coding for ROS1 (50 μg/injection) were administered intramuscularly. In order to increase plasmid immunogenicity, two 25-ms transcutaneous low-voltage electric pulses (amplitude 150 V; interval 300 ms) were applied to the site of injection using the Cliniporator device, which is an instrument that has already been approved for human use, and that has provided striking results in several clinical trials of electrochemotherapy [93,94,95,96,97,98]. The DNA dose, the schedule of administration, and the electroporation protocol were derived from those successfully used to immunize mice against ErbB2 and other antigens [53,54,74,75,76]. The impossibility of predicting whether the electrovaccination using the murine homologous sequence of ROS1 would be able to break immune tolerance, leading to a detectable and effective anti-tumor immune response, spurred us to test the efficacy of both the murine and the human ROS1-coding plasmids (80% homology between human and murine ROS1 sequences). Indeed, it has been clearly demonstrated that heterologous sequences provide the heteroclitic peptides necessary to overcome T cell tolerance [99], and when they share a good degree of homology (ranging from 80% to 95%) with the self sequence [100], an effective cross-reactive immune response can be achieved [53,54,55]. The demonstration of the effectiveness of both DNA vaccines lays the basis for the design of chimeric murine/human ROS1 vaccines, which combine xenogeneic and homologous DNA sequences, as we previously did for other antigens [53,54,55].

When electrovaccination was used to prevent the growth of the transplantable KL-ROS1 cell line, significantly increased survival was observed in the electrovaccinated mROS1 and hROS1 mice, compared to the control mice. The few ROS1 electrovaccinated mice that completely rejected the KL-ROS1 cells developed an effective anti-tumor immune memory and were thus protected from a second challenge of KL-ROS1 tumor cells. Moreover, 25% of mROS1 and 75% of hROS1 electrovaccinated mice were protected from the development of lung metastases. Low levels of specific antibodies were found in ROS1 electrovaccinated mice, and this could justify the absence of ROS1 down-regulation observed in the subcutaneous tumors and lung metastases. However, a significantly higher frequency of γδ T cells and activated NK cells was observed in ROS1 than in control electrovaccinated mice. Furthermore, IFN-γ-secreting CD8^+^ T cells that react against the H-2D^b^ immunodominant ROS1-derived peptide were clearly evident in 50% of hROS1 electrovaccinated mice and barely detectable in just 10% of mROS1 electrovaccinated mice. Central tolerance mechanisms, such as the deletion of the T-cell clone that reacts against the tolerated mROS1 protein, may be responsible for this absence of measurable CD8^+^ T cell reactivity.

The weak anti-ROS1 antibody response, together with the heterogeneity in the induced cellular immune response, highlights the need to improve the vaccination protocol, including the plasmid dose, the number of vaccinations, and the physical parameters used for muscle electroporation. Nevertheless, these data are a proof of concept of the potential efficacy of anti-ROS1 electrovaccination in hampering tumor growth and the metastatic spread of NSCLC. This is even more evident if we consider the results obtained in the spontaneous and aggressive K-Ras^G12D^ model, in which clear disease control was evident in both m and hROS1 electrovaccinated mice. As for wt mice, the involvement of γδ T and activated NK cells can also be envisaged in this setting, as shown by the presence of these cells in the lungs of 30-week-old ROS1 electrovaccinated K-Ras^G12D^ mice. The importance of these cell populations in the lung cancer has recently gained a lot of attention. There are several reports highlighting that properly stimulated γδ T cells can recognize and kill lung cancer cells, while they are altered in advanced NSCLC [101,102,103,104]. Along the same line, several studies have suggest that NK cells have a robust protective role against tumors in the early stages of lung cancer, but that they become dysfunctional in the late stages, allowing tumor evasion to occur [105,106]. Therefore, it is possible that the NK cell activation and γδ T cell expansion that are mediated by ROS1 electrovaccination may prolong their functionality and contribute to lung cancer impairment.

On the basis of these results, we can state that anti-ROS1 DNA electrovaccination has the potential to impair NSCLC progression, even if it is not able to completely block lung carcinogenesis. However, it is important to note that, as in human NSCLC, constitutively active K-Ras expression also induces an immunosuppressive tumor microenvironment [82], which may contribute to the limited effectiveness of our vaccines, in these murine K-Ras^G12D^ models. In particular, we have observed the recruitment of CD8^+^ T cells and M1-type macrophages in the lungs of K-Ras^G12D^ mice in the early stages (10 weeks of age) of NSCLC progression, which hints at the existence of a spontaneous anti-tumor immune response. However, at an advanced stage of the disease (30 weeks of age), a significant rise in the pro-tumorigenic M2-type macrophages percentage was found, which switches the microenvironment to an immunosuppressive one. Moreover, the high expression of PD-L1 in both transplantable and spontaneous K-Ras^G12D^-based models, which is not altered by vaccination, further contributes to dampening the immune response.

The inhibition of the PD1/PD-L1 axis using CIs has provided significant benefits for the treatment of NSCLC patients. However, resistance to treatment occurs frequently. Recent reports have demonstrated that one of the major causes of this resistance is the accumulation of M2-type macrophages at the tumor site [107,108]. For this reason, the concomitant targeting of M2-type macrophages and CI administration has been proposed as a promising strategy that may increase the efficacy of each therapy alone [109]. The availability of the K-Ras^G12D^-based models of ROS1-positive NSCLC, described herein, will allow us to test whether adding ROS1 targeting to this combined treatment would result in an even more effective anti-tumor strategy—one that could ensure the long-lasting and specific activation of the cellular immune response.

As the ROS1 protein is deregulated in a wide range of tumors other than NSCLC, including brain [110,111,112,113], breast [26], liver [114], colon [115], stomach [116], ovarian [117] and oral carcinomas [25], and chronic myelomonocytic leukemia [118], the development of ROS1 electrovaccination may also provide a new therapeutic option for patients affected by these ROS1-expressing cancers.

## 5. Conclusions

In summary, we have demonstrated that a potentially effective anti-ROS1 immune response has been induced using DNA electroporation in a NSCLC murine model, and this may be a valuable proof of concept for further evaluation of the possibility of exploiting this strategy, in combination with others, in the treatment of the wide range of ROS1-expressing tumors.

## Supplementary Materials

The following are available online at https://www.mdpi.com/2076-393X/8/2/166/s1, Supplementary Materials and Methods, Figure S1: ROS1 expression in lung tumors from K-Ras^G12D^ transgenic mice, Figure S2: ROS1 mRNA expression in K-Ras^G12D^/p53^R172HΔg^ (KP) double transgenic mice, Figure S3: Anti-ROS1 humoral immune response induced by ROS1 electrovaccination in wt mice, Figure S4: Vaccine-induced immune response via ROS1 electrovaccination in K-Ras^G12D^ mice.

## References

[1] Wang S., Liu Y., Feng Y., Zhang J., Swinnen J., Li Y., Ni Y. (2019). A Review on Curability of Cancers: More Efforts for Novel Therapeutic Options Are Needed. Cancers.

[2] Rotow J., Bivona T.G. (2017). Understanding and targeting resistance mechanisms in NSCLC. Nat. Rev. Cancer.

[3] Zappa C., Mousa S.A. (2016). Non-small cell lung cancer: Current treatment and future advances. Transl. Lung Cancer.Res..

[4] Sangha R., Price J., Butts C.A. (2010). Adjuvant therapy in non-small cell lung cancer: Current and future directions. Oncologist.

[5] Lemjabbar-Alaoui H., Hassan O.U., Yang Y.W., Buchanan P. (2015). Lung cancer: Biology and treatment options. Biochim. Biophys. Acta.

[6] Jones C.M., Brunelli A., Callister M.E., Franks K.N. (2018). Multimodality Treatment of Advanced Non-small Cell Lung Cancer: Where are we with the Evidence?. Curr. Surg. Rep..

[7] Lim E., Harris G., Patel A., Adachi I., Edmonds L., Song F. (2009). Preoperative versus postoperative chemotherapy in patients with resectable non-small cell lung cancer: Systematic review and indirect comparison meta-analysis of randomized trials. J. Thorac. Oncol..

[8] Felip E., Rosell R., Maestre J.A., Rodriguez-Paniagua J.M., Moran T., Astudillo J., Alonso G., Borro J.M., Gonzalez-Larriba J.L., Torres A. (2010). Preoperative chemotherapy plus surgery versus surgery plus adjuvant chemotherapy versus surgery alone in early-stage non-small-cell lung cancer. J. Clin. Oncol..

[9] Burdett S., Pignon J.P., Tierney J., Tribodet H., Stewart L., Le Pechoux C., Auperin A., Le Chevalier T., Stephens R.J., Arriagada R. (2015). Adjuvant chemotherapy for resected early-stage non-small cell lung cancer. Cochrane Database Syst. Rev..

[10] Chiari R., Sidoni A., Metro G. (2018). Early stage resectable non-small cell lung cancer: Is neoadjuvant immunotherapy the right way forward?. J. Thorac. Dis..

[11] Travis W.D., Brambilla E., Nicholson A.G., Yatabe Y., Austin J.H.M., Beasley M.B., Chirieac L.R., Dacic S., Duhig E., Flieder D.B. (2015). The 2015 World Health Organization Classification of Lung Tumors: Impact of Genetic, Clinical and Radiologic Advances Since the 2004 Classification. J. Thorac. Oncol..

[12] Brahmer J.R., Hammers H., Lipson E.J. (2015). Nivolumab: Targeting PD-1 to bolster antitumor immunity. Future Oncol..

[13] Borghaei H., Paz-Ares L., Horn L., Spigel D.R., Steins M., Ready N.E., Chow L.Q., Vokes E.E., Felip E., Holgado E. (2015). Nivolumab versus Docetaxel in Advanced Nonsquamous Non-Small-Cell Lung Cancer. N. Engl. J. Med..

[14] Herbst R.S., Gandara D.R., Hirsch F.R., Redman M.W., LeBlanc M., Mack P.C., Schwartz L.H., Vokes E., Ramalingam S.S., Bradley J.D. (2015). Lung Master Protocol (Lung-MAP)-A Biomarker-Driven Protocol for Accelerating Development of Therapies for Squamous Cell Lung Cancer: SWOG S1400. Clin. Cancer Res..

[15] Rittmeyer A., Barlesi F., Waterkamp D., Park K., Ciardiello F., von Pawel J., Gadgeel S.M., Hida T., Kowalski D.M., Dols M.C. (2017). Atezolizumab versus docetaxel in patients with previously treated non-small-cell lung cancer (OAK): A phase 3, open-label, multicentre randomised controlled trial. Lancet.

[16] Jenkins R.W., Barbie D.A., Flaherty K.T. (2018). Mechanisms of resistance to immune checkpoint inhibitors. Br. J. Cancer.

[17] Antonia S.J., Villegas A., Daniel D., Vicente D., Murakami S., Hui R., Yokoi T., Chiappori A., Lee K.H., de Wit M. (2017). Durvalumab after Chemoradiotherapy in Stage III Non-Small-Cell Lung Cancer. N. Engl. J. Med..

[18] Li J., He Q., Yu X., Khan K., Weng X., Guan M. (2019). Complete response associated with immune checkpoint inhibitors in advanced non-small-cell lung cancer: A meta-analysis of nine randomized controlled trials. Cancer Manag. Res..

[19] Memon H., Patel B.M. (2019). Immune checkpoint inhibitors in non-small cell lung cancer: A bird’s eye view. Life Sci..

[20] Martins F., Sofiya L., Sykiotis G.P., Lamine F., Maillard M., Fraga M., Shabafrouz K., Ribi C., Cairoli A., Guex-Crosier Y. (2019). Adverse effects of immune-checkpoint inhibitors: Epidemiology, management and surveillance. Nat. Rev. Clin. Oncol..

[21] Kim E.S., Kelly K., Paz-Ares L.G., Garrido P., Jalal S., Mahadevan D., Gutierrez M., Provencio M., Schaefer E., Shaheen M. (2018). Abemaciclib in Combination with Single-Agent Options in Patients with Stage IV Non-Small Cell Lung Cancer: A Phase Ib Study. Clin. Cancer Res..

[22] Shepherd F.A., Rodrigues Pereira J., Ciuleanu T., Tan E.H., Hirsh V., Thongprasert S., Campos D., Maoleekoonpiroj S., Smylie M., Martins R. (2005). Erlotinib in previously treated non-small-cell lung cancer. N. Engl. J. Med..

[23] Iacono D., Chiari R., Metro G., Bennati C., Bellezza G., Cenci M., Ricciuti B., Sidoni A., Baglivo S., Minotti V. (2015). Future options for ALK-positive non-small cell lung cancer. Lung Cancer.

[24] Remon J., Hendriks L.E., Cabrera C., Reguart N., Besse B. (2018). Immunotherapy for oncogenic-driven advanced non-small cell lung cancers: Is the time ripe for a change?. Cancer Treat. Rev..

[25] Shih C.H., Chang Y.J., Huang W.C., Jang T.H., Kung H.J., Wang W.C., Yang M.H., Lin M.C., Huang S.F., Chou S.W. (2017). EZH2-mediated upregulation of ROS1 oncogene promotes oral cancer metastasis. Oncogene.

[26] Tiash S., Chua M.J., Chowdhury E.H. (2016). Knockdown of ROS1 gene sensitizes breast tumor growth to doxorubicin in a syngeneic mouse model. Int. J. Oncol..

[27] Ge W., Han C., Wang J., Zhang Y. (2016). MiR-300 suppresses laryngeal squamous cell carcinoma proliferation and metastasis by targeting ROS1. Am. J. Transl. Res..

[28] Rikova K., Guo A., Zeng Q., Possemato A., Yu J., Haack H., Nardone J., Lee K., Reeves C., Li Y. (2007). Global survey of phosphotyrosine signaling identifies oncogenic kinases in lung cancer. Cell.

[29] Lee H.J., Seol H.S., Kim J.Y., Chun S.M., Suh Y.A., Park Y.S., Kim S.W., Choi C.M., Park S.I., Kim D.K. (2013). ROS1 receptor tyrosine kinase, a druggable target, is frequently overexpressed in non-small cell lung carcinomas via genetic and epigenetic mechanisms. Ann. Surg. Oncol..

[30] Yoshida A., Kohno T., Tsuta K., Wakai S., Arai Y., Shimada Y., Asamura H., Furuta K., Shibata T., Tsuda H. (2013). ROS1-rearranged lung cancer: A clinicopathologic and molecular study of 15 surgical cases. Am. J. Surg. Pathol..

[31] Bergethon K., Shaw A.T., Ou S.H., Katayama R., Lovly C.M., McDonald N.T., Massion P.P., Siwak-Tapp C., Gonzalez A., Fang R. (2012). ROS1 rearrangements define a unique molecular class of lung cancers. J. Clin. Oncol..

[32] Ou S.H., Tan J., Yen Y., Soo R.A. (2012). ROS1 as a ’druggable’ receptor tyrosine kinase: Lessons learned from inhibiting the ALK pathway. Expert Rev. Anticancer Ther..

[33] Go H., Kim D.W., Kim D., Keam B., Kim T.M., Lee S.H., Heo D.S., Bang Y.J., Chung D.H. (2013). Clinicopathologic analysis of ROS1-rearranged non-small-cell lung cancer and proposal of a diagnostic algorithm. J. Thorac. Oncol..

[34] Pan Y., Zhang Y., Li Y., Hu H., Wang L., Li H., Wang R., Ye T., Luo X., Zhang Y. (2014). ALK, ROS1 and RET fusions in 1139 lung adenocarcinomas: A comprehensive study of common and fusion pattern-specific clinicopathologic, histologic and cytologic features. Lung Cancer.

[35] Lin J.J., Ritterhouse L.L., Ali S.M., Bailey M., Schrock A.B., Gainor J.F., Ferris L.A., Mino-Kenudson M., Miller V.A., Iafrate A.J. (2017). ROS1 Fusions Rarely Overlap with Other Oncogenic Drivers in Non-Small Cell Lung Cancer. J. Thorac. Oncol..

[36] Kim M.H., Shim H.S., Kang D.R., Jung J.Y., Lee C.Y., Kim D.J., Lee J.G., Bae M.K., Kim H.R., Lim S.M. (2014). Clinical and prognostic implications of ALK and ROS1 rearrangements in never-smokers with surgically resected lung adenocarcinoma. Lung Cancer.

[37] Kim H.R., Lim S.M., Kim H.J., Hwang S.K., Park J.K., Shin E., Bae M.K., Ou S.H., Wang J., Jewell S.S. (2013). The frequency and impact of ROS1 rearrangement on clinical outcomes in never smokers with lung adenocarcinoma. Ann. Oncol..

[38] Robinson D.R., Wu Y.M., Lin S.F. (2000). The protein tyrosine kinase family of the human genome. Oncogene.

[39] Shaw A.T., Hsu P.P., Awad M.M., Engelman J.A. (2013). Tyrosine kinase gene rearrangements in epithelial malignancies. Nat. Rev. Cancer.

[40] Shaw A.T., Ou S.H., Bang Y.J., Camidge D.R., Solomon B.J., Salgia R., Riely G.J., Varella-Garcia M., Shapiro G.I., Costa D.B. (2014). Crizotinib in ROS1-rearranged non-small-cell lung cancer. N. Engl. J. Med..

[41] Moro-Sibilot D., Cozic N., Perol M., Mazieres J., Otto J., Souquet P.J., Bahleda R., Wislez M., Zalcman G., Guibert S.D. (2019). Crizotinib in c-MET- or ROS1-positive NSCLC: Results of the AcSe phase II trial. Ann. Oncol..

[42] Mazieres J., Zalcman G., Crino L., Biondani P., Barlesi F., Filleron T., Dingemans A.M., Lena H., Monnet I., Rothschild S.I. (2015). Crizotinib therapy for advanced lung adenocarcinoma and a ROS1 rearrangement: Results from the EUROS1 cohort. J. Clin. Oncol..

[43] Kalemkerian G.P., Loo B.W., Akerley W., Attia A., Bassetti M., Boumber Y., Decker R., Dobelbower M.C., Dowlati A., Downey R.J. (2018). NCCN Guidelines Insights: Small Cell Lung Cancer, Version 2.2018. J. Natl. Compr. Canc. Netw..

[44] Guo Y., Cao R., Zhang X., Huang L., Sun L., Zhao J., Ma J., Han C. (2019). Recent Progress in Rare Oncogenic Drivers and Targeted Therapy For Non-Small Cell Lung Cancer. Onco. Targets Ther..

[45] Voena C., Menotti M., Mastini C., Di Giacomo F., Longo D.L., Castella B., Merlo M.E.B., Ambrogio C., Wang Q., Minero V.G. (2015). Efficacy of a Cancer Vaccine against ALK-Rearranged Lung Tumors. Cancer Immunol. Res..

[46] Braso-Maristany F., Griguolo G., Pascual T., Pare L., Nuciforo P., Llombart-Cussac A., Bermejo B., Oliveira M., Morales S., Martinez N. (2020). Phenotypic changes of HER2-positive breast cancer during and after dual HER2 blockade. Nat. Commun..

[47] Silva A.P., Coelho P.V., Anazetti M., Simioni P.U. (2017). Targeted therapies for the treatment of non-small-cell lung cancer: Monoclonal antibodies and biological inhibitors. Hum. Vaccin. Immunother..

[48] Zugazagoitia J., Molina-Pinelo S., Lopez-Rios F., Paz-Ares L. (2017). Biological therapies in nonsmall cell lung cancer. Eur. Respir. J..

[49] Demlova R., Valik D., Obermannova R., ZdraZilova-Dubska L. (2016). The safety of therapeutic monoclonal antibodies: Implications for cancer therapy including immuno-checkpoint inhibitors. Physiol. Res..

[50] Chung C.H., Mirakhur B., Chan E., Le Q.T., Berlin J., Morse M., Murphy B.A., Satinover S.M., Hosen J., Mauro D. (2008). Cetuximab-induced anaphylaxis and IgE specific for galactose-alpha-1,3-galactose. N. Engl. J. Med..

[51] Choi J., Lee S.Y. (2020). Clinical Characteristics and Treatment of Immune-Related Adverse Events of Immune Checkpoint Inhibitors. Immune. Netw..

[52] Cavallo F., Calogero R.A., Forni G. (2007). Are oncoantigens suitable targets for anti-tumour therapy?. Nat. Rev. Cancer.

[53] Quaglino E., Mastini C., Amici A., Marchini C., Iezzi M., Lanzardo S., De Giovanni C., Montani M., Lollini P.L., Masucci G. (2010). A better immune reaction to Erbb-2 tumors is elicited in mice by DNA vaccines encoding rat/human chimeric proteins. Cancer Res..

[54] Jacob J.B., Quaglino E., Radkevich-Brown O., Jones R.F., Piechocki M.P., Reyes J.D., Weise A., Amici A., Wei W.Z. (2010). Combining human and rat sequences in her-2 DNA vaccines blunts immune tolerance and drives antitumor immunity. Cancer Res..

[55] Riccardo F., Bolli E., Macagno M., Arigoni M., Cavallo F., Quaglino E. (2017). Chimeric DNA Vaccines: An Effective Way to Overcome Immune Tolerance. Curr. Top. Microbiol. Immunol..

[56] Lollini P.L., Cavallo F., Nanni P., Quaglino E. (2015). The Promise of Preventive Cancer Vaccines. Vaccines.

[57] Fioretti D., Iurescia S., Fazio V.M., Rinaldi M. (2010). DNA vaccines: Developing new strategies against cancer. J. Biomed. Biotechnol..

[58] Hobernik D., Bros M. (2018). DNA Vaccines-How Far From Clinical Use?. Int. J. Mol. Sci..

[59] Babiuk S., van Drunen Littel-van den Hurk S., Babiuk L.A. (2006). Delivery of DNA vaccines using electroporation. Methods Mol. Med..

[60] Luxembourg A., Hannaman D., Nolan E., Ellefsen B., Nakamura G., Chau L., Tellez O., Little S., Bernard R. (2008). Potentiation of an anthrax DNA vaccine with electroporation. Vaccine.

[61] Riccardo F., Iussich S., Maniscalco L., Lorda Mayayo S., La Rosa G., Arigoni M., De Maria R., Gattino F., Lanzardo S., Lardone E. (2014). CSPG4-specific immunity and survival prolongation in dogs with oral malignant melanoma immunized with human CSPG4 DNA. Clin. Cancer Res..

[62] Aurisicchio L., Mancini R., Ciliberto G. (2013). Cancer vaccination by electro-gene-transfer. Expert Rev. Vaccines.

[63] Rizzuto G., Cappelletti M., Mennuni C., Wiznerowicz M., DeMartis A., Maione D., Ciliberto G., La Monica N., Fattori E. (2000). Gene electrotransfer results in a high-level transduction of rat skeletal muscle and corrects anemia of renal failure. Hum. Gene. Ther..

[64] Cappelletti M., Zampaglione I., Rizzuto G., Ciliberto G., La Monica N., Fattori E. (2003). Gene electro-transfer improves transduction by modifying the fate of intramuscular DNA. J. Gene. Med..

[65] Aurisicchio L., Ciliberto G. (2011). Emerging cancer vaccines: The promise of genetic vectors. Cancers.

[66] Iezzi M., Quaglino E., Amici A., Lollini P.L., Forni G., Cavallo F. (2012). DNA vaccination against oncoantigens: A promise. Oncoimmunology.

[67] Cavallo F., Offringa R., van der Burg S.H., Forni G., Melief C.J. (2006). Vaccination for treatment and prevention of cancer in animal models. Adv. Immunol..

[68] Mir L.M., Moller P.H., Andre F., Gehl J. (2005). Electric pulse-mediated gene delivery to various animal tissues. Adv. Genet..

[69] Mir L.M. (2014). Electroporation-based gene therapy: Recent evolution in the mechanism description and technology developments. Methods Mol. Biol..

[70] Johnson L., Mercer K., Greenbaum D., Bronson R.T., Crowley D., Tuveson D.A., Jacks T. (2001). Somatic activation of the K-ras oncogene causes early onset lung cancer in mice. Nature.

[71] Yushkevich P.A., Piven J., Hazlett H.C., Smith R.G., Ho S., Gee J.C., Gerig G. (2006). User-guided 3D active contour segmentation of anatomical structures: Significantly improved efficiency and reliability. Neuroimage.

[72] Bookout A.L., Mangelsdorf D.J. (2003). Quantitative real-time PCR protocol for analysis of nuclear receptor signaling pathways. Nucl. Recept. Signal..

[73] Macagno M., Bandini S., Stramucci L., Quaglino E., Conti L., Balmas E., Smyth M.J., Lollini P.L., Musiani P., Forni G. (2014). Multiple roles of perforin in hampering ERBB-2 (Her-2/neu) carcinogenesis in transgenic male mice. J. Immunol..

[74] Quaglino E., Riccardo F., Macagno M., Bandini S., Cojoca R., Ercole E., Amici A., Cavallo F. (2011). Chimeric DNA Vaccines against ErbB2+ Carcinomas: From Mice to Humans. Cancers.

[75] Arigoni M., Barutello G., Lanzardo S., Longo D., Aime S., Curcio C., Iezzi M., Zheng Y., Barkefors I., Holmgren L. (2012). A vaccine targeting angiomotin induces an antibody response which alters tumor vessel permeability and hampers the growth of established tumors. Angiogenesis.

[76] Barutello G., Curcio C., Spadaro M., Arigoni M., Trovato R., Bolli E., Zheng Y., Ria F., Quaglino E., Iezzi M. (2015). Antitumor immunization of mothers delays tumor development in cancer-prone offspring. Oncoimmunology.

[77] Rammensee H., Bachmann J., Emmerich N.P., Bachor O.A., Stevanovic S. (1999). SYFPEITHI: Database for MHC ligands and peptide motifs. Immunogenetics.

[78] Milovanovic I.S., Stjepanovic M., Mitrovic D. (2017). Distribution patterns of the metastases of the lung carcinoma in relation to histological type of the primary tumor: An autopsy study. Ann. Thorac. Med..

[79] Sariaydin M., Gunay E., Ulasli S.S., Gunay S., Yavas B.D., Tokyol C., Uysal M., Unlu M. (2016). An unusual metastasis of lung adenocarcinoma: Biceps brachii muscle. Lung India.

[80] Altorki N.K., Markowitz G.J., Gao D., Port J.L., Saxena A., Stiles B., McGraw T., Mittal V. (2019). The lung microenvironment: An important regulator of tumour growth and metastasis. Nat. Rev. Cancer.

[81] Jackute J., Zemaitis M., Pranys D., Sitkauskiene B., Miliauskas S., Vaitkiene S., Sakalauskas R. (2018). Distribution of M1 and M2 macrophages in tumor islets and stroma in relation to prognosis of non-small cell lung cancer. BMC Immunol..

[82] Deng S., Clowers M.J., Velasco W.V., Ramos-Castaneda M., Moghaddam S.J. (2020). Understanding the Complexity of the Tumor Microenvironment in K-ras Mutant Lung Cancer: Finding an Alternative Path to Prevention and Treatment. Front. Oncol..

[83] Chang S., Park H.K., Choi Y.L., Jang S.J. (2019). Interobserver Reproducibility of PD-L1 Biomarker in Non-small Cell Lung Cancer: A Multi-Institutional Study by 27 Pathologists. J. Pathol. Transl. Med..

[84] Riccardo F., Arigoni M., Buson G., Zago E., Iezzi M., Longo D., Carrara M., Fiore A., Nuzzo S., Bicciato S. (2014). Characterization of a genetic mouse model of lung cancer: A promise to identify Non-Small Cell Lung Cancer therapeutic targets and biomarkers. BMC Genom..

[85] Siegel R.L., Miller K.D., Jemal A. (2020). Cancer statistics, 2020. CA Cancer J. Clin..

[86] Dugay F., Llamas-Gutierrez F., Gournay M., Medane S., Mazet F., Chiforeanu D.C., Becker E., Lamy R., Lena H., Rioux-Leclercq N. (2017). Clinicopathological characteristics of ROS1- and RET-rearranged NSCLC in caucasian patients: Data from a cohort of 713 non-squamous NSCLC lacking KRAS/EGFR/HER2/BRAF/PIK3CA/ALK alterations. Oncotarget.

[87] Rossi G., Jocolle G., Conti A., Tiseo M., Zito Marino F., Donati G., Franco R., Bono F., Barbisan F., Facchinetti F. (2017). Detection of ROS1 rearrangement in non-small cell lung cancer: Current and future perspectives. Lung Cancer.

[88] Daoud A., Chu Q.S. (2017). Targeting Novel but Less Common Driver Mutations and Chromosomal Translocations in Advanced Non-Small Cell Lung Cancer. Front. Oncol..

[89] Guisier F., Piton N., Salaun M., Thiberville L. (2019). ROS1-rearranged NSCLC With Secondary Resistance Mutation: Case Report and Current Perspectives. Clin. Lung Cancer.

[90] Sehgal K., Patell R., Rangachari D., Costa D.B. (2018). Targeting ROS1 rearrangements in non-small cell lung cancer with crizotinib and other kinase inhibitors. Transl. Cancer Res..

[91] Katayama R., Gong B., Togashi N., Miyamoto M., Kiga M., Iwasaki S., Kamai Y., Tominaga Y., Takeda Y., Kagoshima Y. (2019). The new-generation selective ROS1/NTRK inhibitor DS-6051b overcomes crizotinib resistant ROS1-G2032R mutation in preclinical models. Nat. Commun..

[92] Conde E., Hernandez S., Martinez R., Angulo B., De Castro J., Collazo-Lorduy A., Jimenez B., Muriel A., Mate J.L., Moran T. (2019). Assessment of a New ROS1 Immunohistochemistry Clone (SP384) for the Identification of ROS1 Rearrangements in Patients with Non-Small Cell Lung Carcinoma: The ROSING Study. J. Thorac. Oncol..

[93] Colombo G.L., Matteo S.D., Mir L.M. (2008). Cost-effectiveness analysis of electrochemotherapy with the Cliniporatortrade mark vs other methods for the control and treatment of cutaneous and subcutaneous tumors. Ther. Clin. Risk Manag..

[94] Schmidt G., Juhasz-Boss I., Solomayer E.F., Herr D. (2014). Electrochemotherapy in Breast Cancer: A Review of References. Geburtshilfe und Frauenheilkunde.

[95] Bianchi G., Campanacci L., Ronchetti M., Donati D. (2016). Electrochemotherapy in the Treatment of Bone Metastases: A Phase II Trial. World J. Surg..

[96] Ricotti F., Giuliodori K., Cataldi I., Campanati A., Ganzetti G., Ricotti G., Offidani A. (2014). Electrochemotherapy: An effective local treatment of cutaneous and subcutaneous melanoma metastases. Dermatol. Ther..

[97] Mir L.M., Morsli N., Garbay J.R., Billard V., Robert C., Marty M. (2003). Electrochemotherapy: A new treatment of solid tumors. J. Exp. Clin. Cancer Res..

[98] Miklavcic D., Sersa G., Brecelj E., Gehl J., Soden D., Bianchi G., Ruggieri P., Rossi C.R., Campana L.G., Jarm T. (2012). Electrochemotherapy: Technological advancements for efficient electroporation-based treatment of internal tumors. Med. Biol. Eng. Comput..

[99] Kianizad K., Marshall L.A., Grinshtein N., Bernard D., Margl R., Cheng S., Beermann F., Wan Y., Bramson J. (2007). Elevated frequencies of self-reactive CD8+ T cells following immunization with a xenoantigen are due to the presence of a heteroclitic CD4+ T-cell helper epitope. Cancer Res..

[100] Cavallo F., Aurisicchio L., Mancini R., Ciliberto G. (2014). Xenogene vaccination in the therapy of cancer. Expert Opin. Biol. Ther..

[101] Yoshida Y., Nakajima J., Wada H., Kakimi K. (2011). gammadelta T-cell immunotherapy for lung cancer. Surg. Today.

[102] Zhao Y., Niu C., Cui J. (2018). Gamma-delta (gammadelta) T cells: Friend or foe in cancer development?. J. Transl. Med..

[103] Sakamoto M., Nakajima J., Murakawa T., Fukami T., Yoshida Y., Murayama T., Takamoto S., Matsushita H., Kakimi K. (2011). Adoptive immunotherapy for advanced non-small cell lung cancer using zoledronate-expanded gammadeltaTcells: A phase I clinical study. J. Immunother..

[104] Bao Y., Guo L., Mo J. (2017). Characterization of gammadelta T cells in patients with non-small cell lung cancer. Oncol. Lett..

[105] Cong J., Wang X., Zheng X., Wang D., Fu B., Sun R., Tian Z., Wei H. (2018). Dysfunction of Natural Killer Cells by FBP1-Induced Inhibition of Glycolysis during Lung Cancer Progression. Cell. Metab..

[106] Cong J., Wei H. (2019). Natural Killer Cells in the Lungs. Front. Immunol..

[107] Bu X., Mahoney K.M., Freeman G.J. (2016). Learning from PD-1 Resistance: New Combination Strategies. Trends Mol. Med..

[108] Arlauckas S.P., Garris C.S., Kohler R.H., Kitaoka M., Cuccarese M.F., Yang K.S., Miller M.A., Carlson J.C., Freeman G.J., Anthony R.M. (2017). In vivo imaging reveals a tumor-associated macrophage-mediated resistance pathway in anti-PD-1 therapy. Sci. Transl. Med..

[109] Lee C., Jeong H., Bae Y., Shin K., Kang S., Kim H., Oh J., Bae H. (2019). Targeting of M2-like tumor-associated macrophages with a melittin-based pro-apoptotic peptide. J. Immunother. Cancer.

[110] Birchmeier C., Sharma S., Wigler M. (1987). Expression and rearrangement of the ROS1 gene in human glioblastoma cells. Proc. Natl. Acad. Sci. USA.

[111] Watkins D., Dion F., Poisson M., Delattre J.Y., Rouleau G.A. (1994). Analysis of oncogene expression in primary human gliomas: Evidence for increased expression of the ros oncogene. Cancer Genet. Cytogenet..

[112] Zhao J.F., Sharma S. (1995). Expression of the ROS1 oncogene for tyrosine receptor kinase in adult human meningiomas. Cancer Genet. Cytogenet..

[113] Jun H.J., Woolfenden S., Coven S., Lane K., Bronson R., Housman D., Charest A. (2009). Epigenetic regulation of c-ROS receptor tyrosine kinase expression in malignant gliomas. Cancer Res..

[114] Gu T.L., Deng X., Huang F., Tucker M., Crosby K., Rimkunas V., Wang Y., Deng G., Zhu L., Tan Z. (2011). Survey of tyrosine kinase signaling reveals ROS kinase fusions in human cholangiocarcinoma. PLoS ONE.

[115] Pietrantonio F., Di Nicolantonio F., Schrock A.B., Lee J., Tejpar S., Sartore-Bianchi A., Hechtman J.F., Christiansen J., Novara L., Tebbutt N. (2017). ALK, ROS1, and NTRK Rearrangements in Metastatic Colorectal Cancer. J. Natl. Cancer Inst..

[116] Lee J., Lee S.E., Kang S.Y., Do I.G., Lee S., Ha S.Y., Cho J., Kang W.K., Jang J., Ou S.H. (2013). Identification of ROS1 rearrangement in gastric adenocarcinoma. Cancer.

[117] Birch A.H., Arcand S.L., Oros K.K., Rahimi K., Watters A.K., Provencher D., Greenwood C.M., Mes-Masson A.M., Tonin P.N. (2011). Chromosome 3 anomalies investigated by genome wide SNP analysis of benign, low malignant potential and low grade ovarian serous tumours. PLoS ONE.

[118] Cilloni D., Carturan S., Bracco E., Campia V., Rosso V., Torti D., Calabrese C., Gaidano V., Niparuck P., Favole A. (2013). Aberrant activation of ROS1 represents a new molecular defect in chronic myelomonocytic leukemia. Leuk. Res..

