# Supplementary material for: Immunization against ROS1 by DNA Electroporation Impairs K-Ras-Driven Lung Adenocarcinomas
Source: Vaccines (Basel). 2020 Apr 6;8(2):166. doi: 10.3390/vaccines8020166 (PMC7349290; doi:10.3390/vaccines8020166)
Supplement: Supplementary file 1 [file vaccines-08-00166-s001.pdf]

Supplementary materials

Supplementary Materials and Methods

Microarray data generation and analysis.

ROS1 mRNA expression level analysis in double transgenic K-Ras^{G12D}/p53$^{R172H\Delta g}$ was based on previously deposited microarray experiments and data processing, described in [84]. Briefly, the dataset included genome-wide gene expression data for the lungs collected from wt and K-Ras^{G12D}/p53$^{R172H\Delta g}$, over a three-point time course (10, 20, and 30 week-old animals), examined using Exon 1.0 ST mouse microarrays, according to the procedure described by the manufacturer (Affymetrix, Santa Clara, California, USA). Arrays have been scanned on an Affymetrix Gene ChIP Scanner 3000 7G and the CEL files were analyzed as previously described [84]. Data were deposited on GEO database: GSE30878.

Assessment of anti-ROS1 humoral response.

Sera from vaccinated wt and Kras^{G12D} mice were collected two weeks after the last electrovaccination. Sera samples were tested by ELISA using the following ROS1 protein fragments: mROS1 fragment (from 29 to 1038 aa) and hROS1 fragment (from 28 to 1042 aa) (all from Genscript). Briefly, 96-well plates (Costar®, Sigma-Aldrich) were coated with 50 ng/well of each protein fragment overnight at 4°C in a bicarbonate coating buffer. Coated plates were then blocked with 10% Newborn Calf Serum (Sigma-Aldrich) in PBS for 2h at 37°C and then incubated with sera 1:100 in 1% blocking buffer for 2h at 37°C. Plates were washed 3 times with a PBS-Tween 0.05% buffer. The horseradish peroxidase (HRP)-conjugated anti-mouse IgG antibody (Sigma-Aldrich; 1:2000 dilution in blocking buffer) was incubated for 1 hour at 37°C. Plates were washed 3 times and chromogenic 3,3′,5,5′-tetramethylbenzidine substrate was added (Sigma-Aldrich). The reaction was stopped by the addition of 2N hydrochloric acid and optical density was measured at 450 nm using a microplate reader (680XR, Bio-Rad, Hercules, California, USA).

For immunofluorescence analysis, NIH-3T3 fibroblasts were seeded on glass slides and transfected using Lipofectamine (ThermoFisher) with the mROS- and hROS-coding plasmids, NIH-3T3 fibroblasts transfected with the empty vector pVAX were used as controls. Forty-eight hours after transfection, cells were fixed in 4% formalin solution and then blocked in 10% BSA (Sigma-Aldrich). Slides were incubated with sera collected from both wt and Kras^{G12D} mice, vaccinated with either pVAX, mROS1 or hROS1 for one hour at room temperature, washed with PBS and incubated with rabbit AlexaFluor488-anti-mouse (Life Technologies, Carlsbad, California, USA). Nuclei were counterstained with DAPI (Sigma-Aldrich). Images were acquired with a Leica TCS-SP5 II confocal microscope and analyzed using LASAF software (Leica).

Supplementary Figures

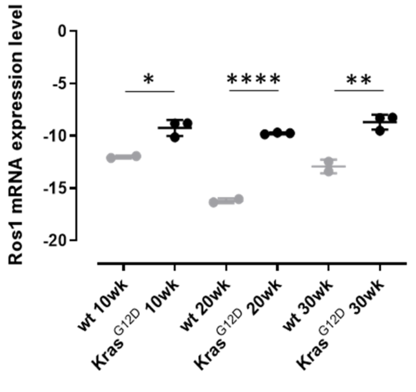

Figure S1. ROS1 expression in lung tumors from K-Ras^{G12D} transgenic mice. qPCR for ROS1 mRNA expression levels in lungs from 10-, 20- and 30-week-old wt and K-Ras^{G12D} mice (N = 2-3). The results are expressed as Delta C$_T$ (-Dct) values between the C$_T$ value of the ROS1 gene and the C$_T$ value of the 18s ribosomal RNA housekeeping gene. Each dot represents the evaluation of the relative mRNA expression level in a single mouse. Statistically significant differences were calculated using the Student's *t*-test: *, 0.02; **, <0.006; ****, <0.0001.

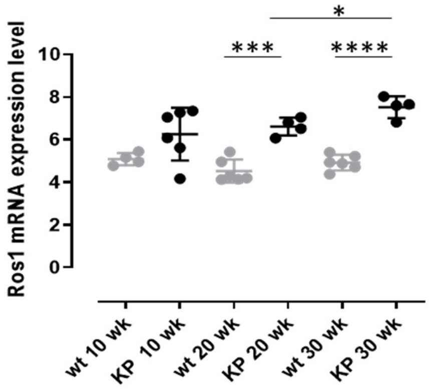

Figure S2. ROS1 mRNA expression in K-Ras^{G12D}/p53$^{R172H\Delta g}$ (KP) double transgenic mice. Microarray analysis of ROS1 mRNA expression in lung samples from wt and KP mice at indicated weeks of age (N = 4-6 mice for each time point) based on previously deposited microarray experiments and data processing described in [84] (GEO accession number GSE30878). Graph represents the mean $\pm$ SD of normalized ROS1 expression levels. Each dot represents the evaluation of gene expression in a single mouse. Statistically significant differences were calculated using the Student's t-test: *, 0.03; ***, <0.0002; ****, <0.0002.

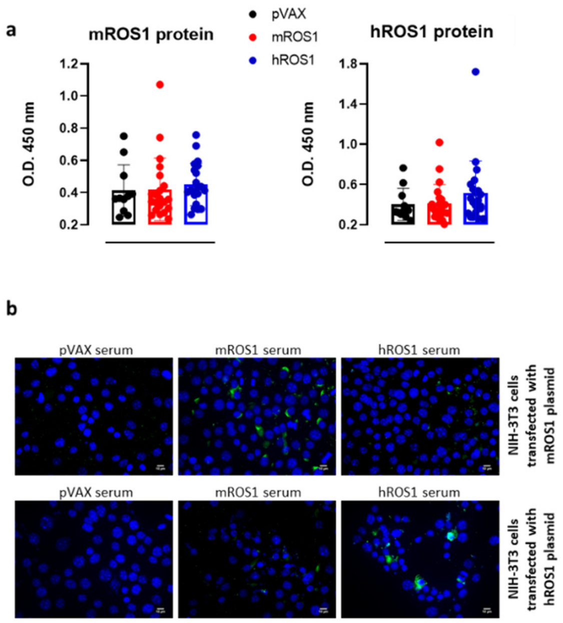

Figure S3. Anti-ROS1 humoral immune response induced by ROS1 electrovaccination in wt mice. Evaluation of vaccine-induced antibody response by ELISA **(a)** and immunofluorescence **(b)**. **(a)** Anti-ROS1 antibody level in pVAX- (black, N = 11), mROS1- (red, N = 21) and hROS1- (blue, N = 21) electrovaccinated K-Ras^{G12D} mice two weeks after the second immunization. Sera from electrovaccinated mice were tested by ELISA against the extracellular portion of the mROS1 (aa 29 to 1038) (left panel) and hROS1 (aa 28 to 1042 and 1043 to 1859) (right panel) proteins. Each dot represents a single mouse serum. Statistically significant differences in the antibody titers were

analyzed using the Student's *t*-test: *, 0.02. **(b)** Representative immunofluorescence images of NIH-3T3 fibroblasts transfected with either the mROS1 or hROS1 coding plasmids stained with pooled sera collected from ROS1- and pVAX-electrovaccinated mice. Results are representative of one of three independent experiments.

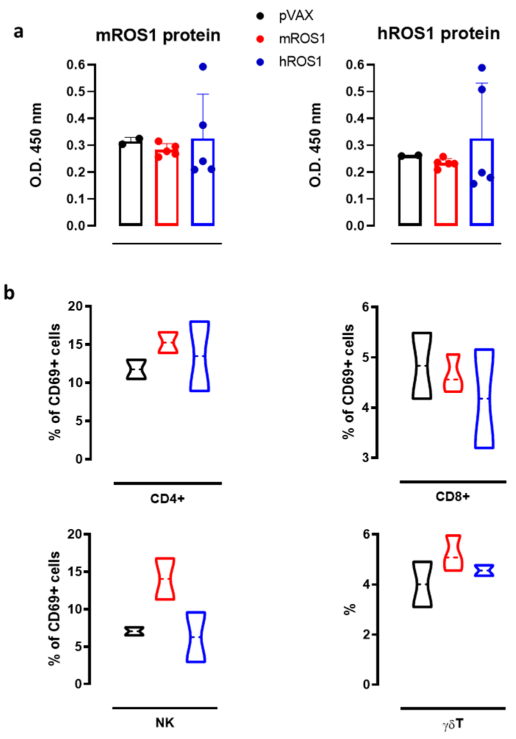

Figure S4. Vaccine-induced immune response via ROS1 electrovaccination in K-Ras^{G12D} mice. **(a)** Anti-ROS1 antibody level in pVAX- (black, N = 2), mROS1- (red, N = 5) and hROS1- (blue, N = 5) electrovaccinated K-Ras^{G12D} mice two weeks after the last immunization. Sera from electrovaccinated mice were tested using ELISA against the extracellular portion of the mROS1 (aa 29 to 1038) (left panel) and hROS1 (aa 28 to 1042 and 1043 to 1859) (right panel) proteins. Each dot represents a single mouse serum. **(b)** Cytofluorimetric analysis of tumors collected from pVAX- (black; N = 3 mice), mROS1- (red; N = 3 mice) and hROS1- (blue; N = 3 mice) electrovaccinated K-Ras^{G12D} mice. CD45$^+$ leukocytes were gated and CD3$^+$ CD4$^+$ CD69$^+$ cells were identified as activated CD4$^+$ T cells, CD3$^+$ CD8$^+$ CD69$^+$ as activated CD8$^+$ T cells, CD3$^-$ CD49b$^+$ CD69$^+$ as activated NK cells and CD3$^+$ γδ$^+$ as γδ T cells. Results are expressed as percentage of positive cells using violin plots, representing the mean and the 95% CI of each group of data.
